# Advances in Defining Ecosystem Functions of the Terrestrial Subsurface Biosphere

**DOI:** 10.3389/fmicb.2022.891528

**Published:** 2022-06-02

**Authors:** D’Arcy R. Meyer-Dombard, Judy Malas

**Affiliations:** Earth and Environmental Sciences, University of Illinois at Chicago, Chicago, IL, United States

**Keywords:** subsurface, continental, dark biosphere, extremophiles, catabolism and anabolism

## Abstract

The subsurface is one of the last remaining ‘uncharted territories’ of Earth and is now accepted as a biosphere in its own right, at least as critical to Earth systems as the surface biosphere. The terrestrial deep biosphere is connected through a thin veneer of Earth’s crust to the surface biosphere, and many subsurface biosphere ecosystems are impacted by surface topography, climate, and near surface groundwater movement and represent a transition zone (at least ephemerally). Delving below this transition zone, we can examine how microbial metabolic functions define a deep terrestrial subsurface. This review provides a survey of the most recent advances in discovering the functional and genomic diversity of the terrestrial subsurface biosphere, how microbes interact with minerals and obtain energy and carbon in the subsurface, and considers adaptations to the presented environmental extremes. We highlight the deepest subsurface studies in deep mines, deep laboratories, and boreholes in crystalline and altered host rock lithologies, with a focus on advances in understanding ecosystem functions in a holistic manner.

## Introduction

### Defining the Terrestrial Subsurface

Defining what is meant by the label “deep biosphere” is not straightforward, as researchers have their own, personal interpretations and there is not a universal community consensus. It’s equally interesting to consider how to define the “surface biosphere.” Is the surface biosphere just the outermost skin of the planet? Does it extend through the entire soil profile or just the first meter (both of which are arbitrary and variable criteria)? Does it extend to the stratosphere? Microorganisms have been identified up to altitudes of 41–77 km in the stratosphere and mesosphere (Wainwright et al., 2004; Griffin, 2008; Pearce et al., 2009; DeLeon-Rodriguez et al., 2013), and are known to influence climate through cloud formation and triggering precipitation events as well as catalyzing atmospheric chemistry (Vaïtilingom et al., 2013; Fröhlich-Nowoisky et al., 2016). No study of the terrestrial deep biosphere has yet reached comparable depths into the subsurface. For comparison, the Chinese Continental Scientific Drilling project (CCSD) recovered continuous cores from depths up to 5,110 m (Zhang et al., 2005; Dai et al., 2021). The lowest reaches of the deep biosphere are currently unknown, but are likely to be governed by physical environmental stressors such as temperature and pressure, as well as the availability and activity of liquid water (Schulze-Makuch et al., 2017; Merino et al., 2019). For example, Dai et al. (2021) found that no microorganisms were detectable in the CCSD cores below 4,850 m depth, estimated to be 137°C. Our ability to investigate fully the depth of the subsurface biosphere is currently hampered by our ability to reach those depths, while providing sufficiently clean samples (see discussions in; Moser et al., 2003; Davidson et al., 2011; Zhong et al., 2018).

Other recent work has reviewed diversity and function in the deep biosphere, from marine, terrestrial, extremophile, and planetary perspectives (e.g., Parkes et al., 2014; Kieft, 2016; Morono and Inagaki, 2016; Colman et al., 2017; Schulze-Makuch et al., 2017; Magnabosco et al., 2018; Merino et al., 2019), each with a different focus. We will consider only the terrestrial deep biosphere, and define this as including bedrock below the soil horizon, at whatever depth that might occur locally. Further, we will synergize recent advances in understanding the metabolic and ecological function of the terrestrial subsurface, highlighting ecosystems where the recent literature showcases efforts to understand the roles of taxa and of microbial communities in the subsurface biosphere.

### Surface Influences on the Terrestrial Subsurface

A necessary topic of discussion, inevitably, is whether the terrestrial subsurface is truly divorced from influence from the surface biosphere. The terrestrial subsurface is hosted within a porous and permeable crust, through which groundwater moves, bringing with it nutrients, carbon, and biomass from the surface biosphere. It is possible that we are not yet technologically capable of reaching depths in the terrestrial subsurface that are truly independent of surface influence - modern or ancient. Many of the biomes commonly considered in the community as “terrestrial subsurface” - by nature of geology and physics - are significantly influenced by surface input. For example, cave biomes have a direct open conduit to the surface if a natural entrance exists, which have the potential to influence metabolic processes within the caves (e.g., Barton and Northup, 2007; Spear et al., 2007; De Waele et al., 2016; D’Angeli et al., 2019; Engel, 2019; Jones and Northup, 2021; Selensky et al., 2021 and references therein). Even without a natural entrance, caves are typically formed in shallow groundwater (Lauritzen, 2018). Another example can be found in the huge body of literature on ecosystems in and under glacial ice (inter- and subglacial ice, respectively), much of which involves discussions of contamination from drilling and sampling protocols, or whether the organisms found represent a paleo-surface signal (e.g., Miteva et al., 2014). The provenance of microorganisms found in interglacial ice is questioned as being endemic vs. sourced from past supraglacial communities or from surrounding biomes (e.g., Zhong et al., 2021). In fact, fluctuations in microbial abundance in interglacial ice has been shown to be correlated with dust load in annual snow (Margesin and Miteva, 2011, and refs. within). While convincing evidence has been presented showing that microorganisms trapped in interglacial ice can actively metabolize (Tung et al., 2005, 2006; Price, 2007; Rhodes et al., 2013; Miteva et al., 2016), these organisms may be recording a history of past climate conditions (Margesin and Miteva, 2011), rather than an endemic modern subsurface biosphere signal.

Another specific biome that is often hailed as a “window” or “portal” to the subsurface is surface springs that have characteristically long residence time or water-rock reaction progress (e.g., hydrothermal or serpentinizing springs). However, the “window” may be opaque and coated with a layer of surface biome. Magnabosco et al. (2014) examined the microbiology of both deep subsurface fracture fluids and related surface springs, and showed that the spring communities were distinct from their fracture fluid counterparts, bringing into question whether springs serve as a reflection of subsurface processes. Further, it has been demonstrated that local climate and topography can directly influence the nutrients and carbon that are present in surface springs. Schubotz et al. (2013) illustrated that the intact polar lipids from biofilms examined from the chemosynthetic zone of two hot springs of different topographic aspect (one seated at the bottom of a wooded slope and the other seated at the top of a mound of hydrothermal deposits) recorded distinct histories of carbon sources and usage. Isotopic analysis of the biomass, DIC, and DOC revealed that the Archaea and Bacteria of the topographically isolated spring recorded a ^13^C enriched signature similar to the ^13^C-DIC (indicating autotrophic growth), while the organisms in the topographically low spring recorded a signature of mixed carbon fixation and heterotrophic metabolisms supported by surface organic carbon. Climate/seasonality can also be a factor in exogenous vs. endogenous carbon use in surface springs as was demonstrated for a system of serpentinizing surface springs in the Philippines (Meyer-Dombard et al., 2019).

**FIGURE 1:**
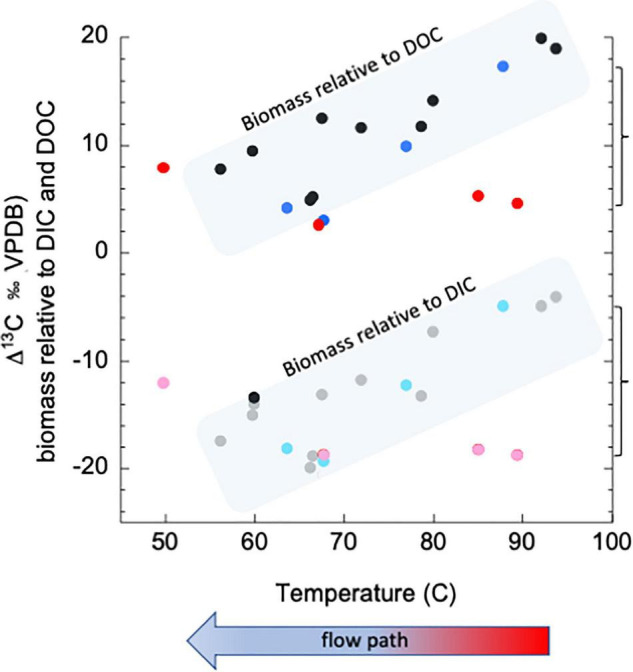
Isotopic composition of carbon in biomass in sediments at “Bison Pool,” Yellowstone National Park, relative to that of dissolved inorganic and organic carbon. Figure shows samples with flow down the outflow channel, with the hottest samples near the source at the right end of the *X*-axis. “Historic,” multi year values are in black and gray points, within blue fields. Values immediately following the precipitation event are in red and pink. Values representing recovery after 8 days are in shades of blue. Methods can be found in Supplementary Material.

Figure 1 further demonstrates the impact of both topography and climate. The data in Figure 1 come from a hydrothermal spring known as “Bison Pool” in Yellowstone National Park, which was serendipitously sampled immediately after a major precipitation event and 8 days following the event. “Bison Pool” is a wide, clear spring, seated nearly level with a surrounding meadow, where one can easily visualize that a window to the subsurface is being presented. Figure 1 shows historic values for ^13^C-biomass (relative to both ^13^C-DIC and ^13^C-DOC, black and gray points, respectively), moving from the source pool brimming with fluids recently in the subsurface to the surface-derived photosynthetic mat downstream. Historic data (encompassed in shaded boxes) show that biomass closest to the high temperature spring source typically incorporates DIC through non-photosynthetic carbon fixation, then trends toward more heterotrophic metabolisms near the 65–70°C section of the outflow channel, and finally returns to carbon fixation through photosynthesis at the end of the outflow (also demonstrated in Swingley et al., 2012). Following the precipitation event, we observed that overland flow was washing the soil and animal excrement from the ground into the pool source, turning the normally clear fluid a turbid brown. The biofilms in the hottest parts of the outflow channel immediately after the rain event (red and pink points) are depleted by as much as ∼5–15‰ relative to both DIC and DOC, indicating an incorporation of more surface derived organic carbon than is typical (compared to “historic” data, note brackets at right). Eight days following the event (light and dark blue points), the biomass had returned to “normal” values, indicating a return to incorporation of DIC in the hottest portions of the pool - the areas that are typically thought of as being “windows” to the subsurface.

This vignette reminds us that the subsurface biosphere, by definition, intersects the surface biosphere. Springs, caves, glaciers, and even aquifers are subsurface biomes that have at least intermittent connection to the surface biosphere. For example, near surface aquifers (e.g., Boyd et al., 2007; Anantharman et al., 2016) and boreholes into sedimentary units (e.g., Katsuyama et al., 2013; Dong et al., 2014; Frank et al., 2016; Hu et al., 2016; Thieringer et al., 2021) may have frequent contact with water, nutrients, and carbon that originated in the surface biome due to high permeability and porosity of host rock.

The degree of connection between the surface and subsurface at any given study area is often a subject of discussion or even concern, if the purpose of the study is to identify a true subsurface signal. While we posit that the connection between the subsurface and surface biospheres is equally as interesting as identifying a subsurface biosphere that is independent of all surface influence, to narrow the scope of this review we will focus on investigations of boreholes, deep mines, and subsurface laboratories, which we feel are the best, current examples of locations with minimized surface influence.

### The Environment of the Deep Terrestrial Subsurface

Environmental conditions, modulators, and nutrient flow in the subsurface are necessarily directed by the type of bedrock that hosts the specific subsurface ecosystem. For example, porosity and permeability of the host rock will partly determine the access the system has to water, nutrients, and carbon sources that moving water may bring with it. Further, these properties may allow direct connection of the surface and subsurface biospheres (e.g., aquifers contaminated by surface sources). In general, environmental conditions that are likely ubiquitous in the terrestrial subsurface biosphere include anaerobicity or very low oxygen concentrations, high pressure, and nutrient limitation. Some locations may also host fluids of higher temperature and salinity than similar surface environments. Several studies have remarked on the probability that most deep subsurface microorganisms are likely surface-attached, or have demonstrated that biofilms form *in situ* on fracture surfaces, and one estimate suggests that biofilms make up 20–80% of the total subsurface biomass (Jägevall et al., 2011; Magnabosco et al., 2018; Flemming and Wuertz, 2019). These observations have direct implications for estimates of subsurface biomass and functioning of ecosystems in the host rock (e.g., Colman et al., 2016).

Regardless of aspect, the host rock water interaction is a key component of defining all terrestrial subsurface ecosystems. Except in cases where organic compounds from the surface biosphere are delivered to the deeper subsurface biosphere, energy and nutrients for life in the subsurface are defined by the interaction between the host rock and water. This interaction is also influenced by surface variables such as local climate and topography, which influence groundwater recharge and flow. Metabolisms that might take place in the subsurface are then guided by these environmental variables and the host rock mineralogy. Further, there is evidence that some water rock interactions may limit the ability for a subsurface community to survive. For example, groundwater interaction with evaporite sequences in the Boulby Mine subsurface were shown to produce brines, some of which resisted cultivation and DNA extraction efforts (Payler et al., 2019). This example illuminates and emphasizes the dependence of the terrestrial subsurface biosphere on the minerals present and groundwater origin and flow path. The systems examined here are hosted in a variety of parent rock and geologic settings, including from volcanic, metamorphic, and ultramafic assemblages, which have implications for resulting geochemistry, energy availability, and functional/taxonomic diversity in the subsurface ecosystem (Boyd et al., 2007; Magnabosco et al., 2018; Leong and Shock, 2020).

## Exploring the Terrestrial Deep Biosphere

Arguably, the most direct access to the deep subsurface with minimal surface influence is in places where we have physically entered the ecosystem *via* mining. The deep subsurface community has been making best use of economic mines for decades now, providing literature that explores the microbial ecosystems in solid rock and pore fluids. Some studies further drill directly into the walls of previously cut mine shafts and tunnels, to obtain more pristine materials. Several underground laboratories and observatories have been established, and modern DNA sequencing technologies are providing fresh perspectives. The deep subsurface has also been frequently accessed *via* drilling of boreholes directly into substrata. The advantage of removing material by drilling (whether from the surface or from within a mine), is that experimental and analytical apparatus can be inserted. Several studies mentioned in the following paragraphs utilize this technique, delivering short and long term incubation experiments and continuous fluid collection.

It must also be recognized that even these best examples of accessing the deeper terrestrial biosphere are not completely without interaction from surface environments. The act of mining by nature exposes the subsurface to the surface atmosphere and surface fluids. Drilling places materials downhole that aren’t indigenous and may not be fully sterile. By necessity, a major focus of working in deep biosphere settings involves understanding, managing, and mitigating contamination. Creative solutions have been developed, particularly to address situations where cell numbers can be vanishingly small (e.g., Zhong et al., 2018, 2021), that have driven innovation in technology and technique (comprehensive descriptions in Hodgson et al., 2016; Kieft, 2016; Morono and Inagaki, 2016). For example, hot water drilling techniques were developed to clean access sediment cores beneath subglacial lake Whillans (Christner et al., 2014; Hodgson et al., 2016) and other technological advances are needed for fast moving ice shelves, or for work in remote locations (Hodgson et al., 2016). The studies highlighted below have shown reasonable attempts at ensuring a lack of surface signal, or defining where potential surface signal is found.

### Deep Mines and Laboratories

We start our discussion of metabolism and ecological function in the terrestrial deep subsurface by reviewing recent work that has emerged from the world’s deep mines and laboratories. The terrestrial subsurface community has long recognized that such direct access is invaluable for microbiological investigations. Leading the way in underground microbiological research are permanently established laboratory sites such as the Kidd Creek Mine Observatory, Äspö Hard Rock Laboratory, Olkiluoto Spent Nuclear Fuel tunnel (ONKALO), Mizunami Underground Research Laboratory, Mont Terri Rock Laboratory, the Boulby Underground Laboratory, and the Sanford Underground Research Laboratory (Stroes-Gascoyne et al., 2007; Fukuda et al., 2010; Pedersen, 2012, 2013; Osburn et al., 2014; Cockell et al., 2019; Lollar et al., 2019). Adding to this wealth of data are studies from retired and working mine locations, which provide additional opportunities for examining the limits of life in the subsurface (e.g., Chivian et al., 2008; Bagnoud et al., 2016; Payler et al., 2019).

#### Fennoscandian Shield Deep Laboratories: Aspö and Pyhäsalmi

One well studied site is the Äspö Hard Rock Laboratory (HRL) in Sweden, which has been established as a deep laboratory for decades (Kotelnikova and Pedersen, 1998). Äspö HRL is a coastal site, situated in the porphyritic granite-granodiorite of the Fennoscandian Shield, and intrusion ages (U-Pb) suggest ages between 1,760 and 1840 Ma (Johansson, 1988). Groundwater in the Äspö HRL is a mix of meteoric and seawater origins, depending on the depth of the boreholes (Kotelnikova and Pedersen, 1998). Fracture fluids are neutral pH, are reduced and depleted in dissolved oxygen and nitrate, but carry measurable amounts of DOC and bicarbonate, as well as sulfate (Kotelnikova and Pedersen, 1998; Wu et al., 2016).

Interest in a “hydrogen driven” subsurface biosphere and the determination that biogenic methane is produced in boreholes in the Fennoscandian Shield (Sherwood Lollar et al., 1993; Kotelnikova and Pedersen, 1998) encouraged an early focus on methanogenic and acetogenic organisms at the Äspö HRL (e.g., Pedersen, 1993; Stevens and McKinley, 1995). It was predicted that groundwaters in Äspö HRL provide an anaerobic and oligotrophic environment that could support nitrate, ferric iron, sulfate, and manganese reducing organisms (Hallbeck and Pedersen, 2012; Pedersen, 2013; Ionescu et al., 2015). Early work at Äspö HRL sites was focused on culture dependent methods. This included the use of a system that recirculates fracture fluids through refrigerated flow cell “cabinets” and then back through the fracture, while maintaining the 3.15 MPa *in situ* fluid pressure within a microbiology dedicated laboratory (“MICROBE,” at 447 m depth) (Hallbeck and Pedersen, 2008). The flow cells allow experimentation with fracture water as well as on surfaces such as glass slides or mineral coupons.

Microorganisms capable of nitrate, sulfate, iron, and manganese reduction, methanogenesis, and acetogenesis were found in groundwater at the MICROBE site in densities ∼ 8 × 10^7^ cells/ml (Hallbeck and Pedersen, 2008), and ^14^C and ^3^H labeled substrates were incorporated into biomass, demonstrating autotrophic and heterotrophic metabolic capabilities in a related location (Pedersen and Ekendahl, 1992; Ekendahl and Pedersen, 1994). Novel methanogens and sulfate reducing bacteria have been described from the Äspö HRL (Kotelnikova et al., 1998; Kotelnikova and Pedersen, 1998; Motamedi and Pedersen, 1998), and it has been suggested that this site is largely supported by hydrogen, a strong electron donor (Pedersen, 2012). The flow cell cabinets were used to experiment with *in situ* batch cultures, with hydrogen or acetate additives, to demonstrate that hydrogen can support a slow but sustainable level of microbial activity in Äspö HRL groundwaters (Pedersen, 2012).

Wu et al. (2016) further queried the planktonic communities in groundwaters of the Äspö HRL to determine the genetic potential for metabolic schema. This work examined three fluids of different age and origin. The resulting metagenomic analysis revealed that the fracture water of modern, marine origin likely represents a more surface impacted fluid. Populations in the modern, marine fracture fluids were shown to be able to primarily ferment organic carbon, and potentially couple sulfide oxidation to nitrate reduction and fix carbon with the reductive pentose phosphate cycle (no other C-fixation pathways were represented). Geochemical data supports these conclusions; fracture water is depleted in nitrate but features measurable ammonium, as well as low sulfide but abundant sulfate. The metagenome from the older, saline fracture waters suggested, again, a large dependence on organic carbon, as well as nitrate and sulfur reduction, denitrification, and carbon and nitrogen fixation. Further, despite the previous demonstrations that a wide range of hydrogen supported, reducing metabolic pathways could be stimulated through enrichment, no genetic signal was found to suggest that these are dominant metabolic functions in the Äspö HRL groundwaters.

A second mine site in the Fennoscandian Shield offers an opportunity to explore different fracture water host rock relationships than the Äspö HRL mine site. In contrast to Äspö, the Pyhäsalmi mine in Finland is seated in Paleoproterozoic volcanogenic massive sulfides, where the lower mine stratigraphy is composed of felsic, tuffaceous volcanites and the upper mine is seated in mafic lavas, breccias, and pyroclastics (Miettinen et al., 2015). Bomberg et al. (2019) explored fracture waters in the upper mine, between 240–600 m depth using enrichments (focusing on iron based metabolisms) coupled with taxonomic diversity analysis, following up on a previous study by Kay et al. (2014). At these depths, the freshly exposed surfaces allow oxidation of the host rock, weathering the minerals and impacting the fracture fluids. Fracture fluids were at pH 1.4–2.3, reminiscent of acid mine drainage systems. Concurrently, the fluids contained high concentrations of the analyzed metals (such as Fe, Mn, Cu, Zn, Al), and both sulfate and sulfide. This study site presents a good example of a deep subsurface site that has been highly impacted by the surface biosphere, and illustrates the plasticity of the resident microbial communities. Enrichments targeted aerobic ferrous iron oxidizers and ferric iron reducers, and results showed a high microbial diversity. Dominant members differed with depth, and *Acidithiobacillus* and *Leptospirillum* dominated at shallower depth while *Ferrovum* and *Metallibacter* were the dominating bacterial genera at 600 m. Species of the heterotrophic genera, *Acidiphilum*, were detected in all samples and enrichments.

Purkamo et al. (2020) investigated the same mine using metagenomics, but at 2.4 km where the fracture fluids were alkaline and reduced, more typical of a deep subsurface mine fluid with less surface influence. Here, 96% of the 16S rRNA marker genes sequenced at this site were gamma and alpha Proteobacteria, and archaeal sequences were essentially not retrieved - it was estimated that only ∼50% of the bacterial richness was captured from these low biomass fluids. Notably, marker genes for sulfate reduction and methanogenesis were not detected. However, the complete dissimilatory nitrate reduction pathway and reductive pentose phosphate pathways were identified (KEGG reconstructions). Carbon cycling was primarily heterotrophic, much like the Äspö sites described above, and functional predictions indicated that chemoheterotrophy was a key ecological function in this environment, with indication that heterotrophy could be coupled with sulfate or sulfur respiration.

Together, these investigations of the deep biosphere seated in the Fennoscandian shield bedrocks point to a low biomass ecosystem that is driven by chemoheterotrophy, nitrate reduction, and reduction of sulfur compounds. Carbon fixation may occur through the reductive pentose phosphate cycle. However, the evidence of a hydrogen-driven ecosystem, with emphasis on methanogenesis, acetogenesis, and sulfate reduction is lacking in the most recent metagenomic-driven studies (Wu et al., 2016; Purkamo et al., 2020), despite earlier successes with targeted enrichments. Future work may elucidate the role of these metabolic options in these sites as supportive, rather than dominant, as in Lau et al. (2016). Further, while each deep mine highlighted here hosts fluids that are clearly impacted by the surface biosphere, truer signals of the subsurface biosphere were apparent at depth at both Äspö HRL and Pyhäsalmi.

#### Sanford Underground Research Facility and Deep Mine Microbial Observatory

At a depth intermediate between the Fennoscandian Shield Äspö HRL and Pyhäsalmi mine locations, the Sanford Underground Research Facility (SURF) in the former Homestake Gold Mine (South Dakota, United States) is home to the Deep Mine Microbial Observatory (DeMMO). The mine is seated in Paleoproterozoic metasediments that are iron rich, and the complex geologic history documents oceanic volcanisms and subsequent marine infilling (Osburn et al., 2019). A summary of primary minerals present in each of the major units at SURF can be found in Casar et al. (2021a), Table 1. Numerous boreholes, manifolds, and pools were available within the mine for sampling prior to the establishment of the DeMMO and new boreholes were drilled (horizontally) at the 1.48 km depth for fresh rock analyses (e.g., Osburn et al., 2014; Momper et al., 2017a). Fresh holes in differing lithologies and fluid flow rates were produced in 2016, some of which were fitted with custom made expandable packers (Osburn et al., 2019). The SURF laboratory and DeMMO locations have produced several studies dedicated to understanding metabolism and microbe-mineral interaction in the deep terrestrial subsurface.

**TABLE 1:** Reactions considered in estimations of Gibbs Free Energy of Reaction shown in Figure 2. Aqueous forms were used for O_2_, CH_4_, H_2_, N_2_.

	Reaction	Electron acceptor	e^–^/rxn
1	O_2_ + 2H_2_ ↔ 2H_2_O	O_2_	4
2	4Fe^+2^ + O_2_ + 6H_2_O ↔ 4FeOOH + 8H^+^	O_2_	4
3	2NO_3_^–^ + 2H^+^ + 5H_2_ ↔ N_2_ + 6H_2_O	NO_3_^–^	10
4	8NO_3_^–^ + 3H^+^ + 5HS^–^ ↔ 5SO_4_^–2^ + 4N_2_ + 4H_2_O	NO_3_^–^	40
5	NO_3_^–^ + H^+^ + CH_4_ ↔ HCO_3_^–^ + NH_4_^+^	NO_3_^–^	8
6	SO_4_^–2^ + H^+^ + 4H_2_ ↔ HS^–^ + 4H_2_O	SO_4_^–2^	8
7	SO_4_^–2^ + CH_4_ ↔ HCO_3_^–^ + HS^–^ + H_2_O	SO_4_^–2^	8
8	4SO_4_^–2^ + 5H^+^ + 3CH_4_ ↔ 3HCO_3_^–^ + 4S^0^ + 7H_2_O	SO_4_^–2^	24
9	acetate + SO_4_^–2^ ↔ 2HCO_3_^–^ + HS^–^	SO_4_^–2^	8
10	S^0^ + H_2_ ↔ H^+^ + HS^–^	S^0^	2
11	HCO_3_^–^ + H^+^ + 4H_2_ ↔ CH_4_ + 3H_2_O	HCO_3_^–^	8
12	4H_2_ + H^+^ + 2HCO_3_^–^ ↔ acetate + 4H_2_O	HCO_3_^–^	4
13	8FeOOH + acetate + 15H^+^ ↔ 8Fe^+2^ + 2HCO_3_^–^ + 12H_2_O	FeOOH	8
14	Fe_2_O_3_ + H_2_ + 4H^+^ ↔ 2Fe^+2^ + 3H_2_O	Fe_2_O_3_	2
15	Fe_3_O_4_ + H_2_ + 6H^+^ ↔ 3Fe^+2^ + 4H_2_O	Fe_3_O_4_	2

Given the complex geologic history of the host rock, it is no surprise that the fracture fluids are geochemically diverse at different DeMMO locations. The concentration of cations in six sites of different depths (and thus different host formations) varies considerably from site to site, even while anions are relatively consistent (high sulfate with low carbonate, or moderate sulfate and carbonate) (Osburn et al., 2019). Redox sensitive chemical species are highly variable, and provide a wide range of energetic options for metabolic pathways (Osburn et al., 2014). Fluid chemistry is extremely consistent over time, suggesting that microbial communities may enjoy environmental stability. Further, it has been demonstrated repeatedly that the fracture fluids in DeMMO are distinct chemically and microbiologically from controls and service waters used in the mine (Osburn et al., 2014, 2019; Casar et al., 2020).

Surveys of diversity at various sites (∼240 m–1.48 km) reveal that among the boreholes, biofilms, pools, and manifolds, Bacteria dominate the communities with Archaea representing at most 5% of the populations. Site fluids differ in dominant taxa. For example, at ∼250 m depth, where fluids are only moderately reducing and have high concentrations of ferrous iron (up to 2.3 mg/L), the communities are dominated by *Ca. Omnitrophica* and various unclassified taxa. In contrast, the 600 m depth fluid is reduced and very rich in both ferrous iron and sulfate (2.8 and 1,800 mg/L, respectively), and is dominated by Betaproteobacteria and *Nitrospirae*. Finally, at 1.5 km, the fluids contain high concentrations of sulfate and methane and primarily host Deltaproteobacteria and *Firmicutes* (Osburn et al., 2014). Fracture fluids appear to support a much higher diversity and richness of organisms than rock-hosted samples (Momper et al., 2017a; Casar et al., 2020, 2021a,b).

A series of investigations fueled by metagenome and experimental methodologies paired with extensive catabolic modeling efforts have elucidated the genetic and metabolic potential of communities in DeMMO sites. At the time of this writing, 22 high quality “MAGs” (metagenome assembled genomes) that are > 90% complete were recovered from fluids at the 1.48 km DeMMO site and other fluids in the SURF complex (Momper et al., 2017b). The reductive Acetyl Co-A pathway was dominant, with a few select MAGS hosting complete pathways for the 3-hydroxypropionate/4-hydroxybutyrate and reductive pentose phosphate cycles (Momper et al., 2017b). The dominance of the reductive Acetyl-CoA pathway has also been noted in several other deep terrestrial biosphere sites (e.g., Nyyssönen et al., 2014; Lau et al., 2016; Magnabosco et al., 2016; Rempfert et al., 2017). The genetic capacity for methanogenesis and sulfate/sulfite reduction were identified in one and thirteen (out of 74) MAGS, respectively. Marker genes for nitrate reduction (both cytoplasmic and periplasmic) were present in several MAGs. However, while all genes needed for denitrification were identified, none were all present together in one MAG. Likewise, evidence for the genetic capacity for CO, thiosulfate, sulfur or sulfide, or iron redox was not found in this particular study (but read on!). These suggested metabolic functions of Bacteria in the fracture fluids agree well with the calculated energy density for these metabolic strategies (Osburn et al., 2014).

Despite the apparent lack of genetic markers for iron-based metabolism in DeMMO fluids, taxonomy and catabolic modeling studies suggested that iron cycling should likely play a large role in ecosystem dynamics. Experiments demonstrated growth of biofilms on surfaces of minerals bearing Fe (as well as S, Ti, Mn), spatially targeting these metals (Casar et al., 2020, 2021a). Biofilms on native rock coupons were enriched in taxa putatively capable of pyrite oxidation with nitrate, a thermodynamically favorable catabolic option that releases ferrous iron and sulfate. In light of this, both new and previously published metagenomes were specifically annotated with an Fe-centric pipeline. Casar et al. (2021b) found that the genetic capacity for iron oxidation existed at all sites analyzed, however, the specific genes present varied from site to site, apparently in accordance with local fracture fluid geochemistry. For example, the shallower, less reducing fluids contained the highest abundances of Cyc2 cluster 1, characteristic of neutrophilic iron oxidizers such as *Gallionella* (identified in MAGs). In general, Cyc2 was identified from most sites, affiliated with different families of Bacteria, demonstrating a wide spread genetic potential for iron oxidation. In addition, genes encoding for iron reduction (*OmcS*, *OmcZ*, and DFE) were abundant at some sites, suggesting that biofilm forming taxa identified in the MAGs can carry out iron/metal reduction on the surface of DeMMO minerals. These lines of genetic and modeling evidence point to iron cycling as a key metabolic function in the subsurface at this site.

These studies reveal a metabolic landscape in the subsurface at SURF. Modeling predicted modes of catabolism that should be favorable in the fluids, namely that the oxidation of sulfur, sulfide, ferrous iron, ammonium, and the reduction of manganese oxides are strong potential *in situ* energy sources, and that the energy density is highest at most sites examined using S^0^, HS^–^, NH_4_^+^, Fe^+2^, and Mn^+2^ as electron donors, but lower using CH_4_, CO, and H_2_. Generally, the oxidation of S^0^, HS^–^, NH_4_^+^, and Fe^+2^ yields large reservoirs of energy in all fracture fluids (per kg of H_2_O), regardless of the electron acceptor (Osburn et al., 2014; Rowe et al., 2020; Casar et al., 2021b). Hydrogen oxidation, methanogenesis, sulfate reduction, and aqueous iron reduction were found to yield little to no energy when normalized as energy density (Osburn et al., 2014). Metabolism associated with mineral surfaces, specifically of S, Fe, Ti, and Mn minerals (Casar et al., 2021a) was predicted to drive biofilm growth on solid rock. The reduction of oxidized iron minerals, such as ferrihydrite, magnetite, goethite, hematite, and lepidocrocite, using HS^–^ and the oxidation of siderite and pyrite with NO_3_^–^ were shown to be particularly energy dense catabolic options for mineral biofilms, while planktonic communities have options for oxidation of aqueous iron (with NO_3_^–^) and reduction of particulate ferrihydrite (with HS^–^) (Casar et al., 2021b). Combined with experimental and metagenomic evidence, this evidence of likely catabolic processes in the subsurface is particularly convincing.

#### South African Mines

The mines in the Witwatersrand represent deep access to Archaean rocks and some of the deepest studies of the terrestrial subsurface biosphere have emerged from this region. This basin is made up of several supergroups of different host rock lithologies; namely, the 2.5 Ga Transvall Supergroup (dolomites, banded iron formation and volcanics), the 2.7 Ga Ventersdorp Supergroup (basaltic), and the 2.9 Ga Witwatersrand Supergroup (quartzite and shale). Below these lies a 3.4–3.0 Ga metamorphic and igneous complex basement, including granite, amphibolite, and gneiss (Nicolaysen et al., 1981). Two types of fracture waters in this region have been identified, ranging in age between 1.5–23 Ma (Lippmann-Pipke et al., 2011). At 0.8–2 km depths, in the Beatrix, Masimong, Merriespruit and Joel mines, paleo-meteoric waters contain hydrocarbon gasses of primarily microbial origin and very little H_2_ gas (Ward et al., 2004; Lippmann-Pipke et al., 2011). Fluids at 2.7–3.6 km in the Kloof, Driefontein, Mponeng, and TauTona mines are saline and host high levels of H_2_ gas (Sherwood Lollar et al., 2007; Lippmann-Pipke et al., 2011). Ancient waters, formed > 2 Ga, enter the fracture fluid by intersecting with fluid inclusions (Lippmann-Pipke et al., 2011). The “self-sufficient,” deep subsurface *Candidatus Desulforudis audaxviator* (Lin et al., 2006a; Chivian et al., 2008) was identified from fracture fluids at 2.8 km depth in the Mponeng mine, with some of the highest contributions of the > 2 Ga fluids among all the fluids investigated by Lippmann-Pipke et al. (2011). Arguably, these mines represent some of the best access to the deep and ancient subsurface. Indications of temporal changes in fracture fluid composition have implications for long term community variability (Magnabosco et al., 2018).

Our discussion will include studies from four of the aforementioned mines, beginning at the shallower depths. Some early indications that shallower fracture fluids (e.g., Beatrix mine) are more microbially diverse than fluids from more saline fluids deeper in the basin (Lin et al., 2006b) may not hold up to scrutiny using modern sequencing methodologies. For example, when Magnabosco et al. (2014) compared samples from six different deep mines in the region, including Beatrix, Driefontein, and TauTona gold mines in the Witwatersrand, they found no statistical difference in the richness or evenness of taxa in fracture fluids between the shallower Beatrix site and the deeper mine sites, and 220/874 total taxa observed in the pooled sample set were shared between all locations. Using high-throughput DNA sequencing to study taxonomic diversity in these deep mines increased the number of identified taxa from previous studies (from 243 taxa), possibly by further identifying rare taxa (Magnabosco et al., 2014).

The Beatrix mine is at the southwestern edge of the Witwatersrand Basin where both the Transvaal Supergroup and Pilanesberg dikes are absent and the sequence is overlain by sedimentary strata. As such, the mine’s fracture fluids flow through quartzite or between the contact of the quartzites and the sedimentary units. Simkus et al. (2016) used carbon isotopic analysis to reveal that fracture fluid biomass incorporated carbon from both biogenic methane and DIC derived from methane oxidation. A recent study by Magnabosco et al. (2018) focused on the methane oxidizing community in these 1.3 km deep fracture fluids, over both long and short time scales. Over a 2.5 year period, the Eh, sulfate, nitrate, and hydrogen concentrations shifted considerably, as did the abundances of specific Archaea and Bacteria. In particular, the methane oxidizing community shifted from a ANME-1- to a *Ca. M. nitroreducens-* dominated community over several years (based on abundances of *mcrA*, *mmo*, and 16S rRNA genes). These shifts correlated to an increase of nitrate over the same time period, and experiments using ^13^CH_4_ coupled to NO_3_^–^ as an electron acceptor resulted in an increase of ^13^CO_2_, suggesting fluctuations of the two populations with shifting electron acceptors (Magnabosco et al., 2018). Metagenomic analysis showed that methanogens and methanotrophs represented < 5% of the total population, signaling that organisms at this depth are utilizing carbon processed by a very small fraction of the community (Simkus et al., 2016).

Extensive literature exists for the deeper mines in the Witwatersrand Basin, which includes the Driefontein, Mponeng, Kloof, and TauTona mines. At intermediate depths, (2.6–2.8 km), solid surfaces, such as crushed rock substrate and fracture surfaces were demonstrated to host biofilms on both basalt and quartzite exposed to fracture fluids (Baker et al., 2003; Wanger et al., 2006). Established *Desulfotomaculum spp*. could be induced to reduce sulfate on most probable number (MPN) plates enriched with lactate or hydrogen (Baker et al., 2003). Lin et al. (2006a) described a community from a 2.8 km depth fracture fluid in the Ventersdorp basalt (Mponeng mine) that was 88% composed of *Desulfotomaculum spp*. (based on molecular cloning methods), and isotopes of sulfur indicated potential microbial sulfate reduction. Chivian et al. (2008) further supported these works by assembling the genome of *Ca. Desulforudis audaxviator*, also from a fracture fluid at 2.8 km in the Mponeng mine, where communities are overwhelmingly dominated by this taxon. This sporulating, sulfate-reducing, chemoautotrophic thermophile likely fixes its own carbon and possibly nitrogen (Chivian et al., 2008), and is a dominant member of many fluids sampled below 1.5 km in the Witwatersrand Basin (e.g., Moser et al., 2003, 2005; Gihring et al., 2006; Lin et al., 2006a). These approaches show that sulfate reduction is likely an important catabolic path in the ∼2.7 km fluids passing through basalt and quartzite hot rock.

Magnabosco et al. (2016) and Simkus et al. (2016) investigated possible avenues for carbon cycling at > 3 km depths. Using metagenomic, isotopic, and “energy flux” analysis, they determined a variety of potential sources of anabolism, and evaluated the likelihood of several catabolic pathways that could support cellular growth. In the deeper, 3 km TauTona mine borehole “TT107,” protein encoding genes (PEGs) for six different carbon fixation pathways were found in the fracture fluids, and the reductive acetyl-CoA pathway was the best represented among the 289 total identified taxa, dominated by Firmicutes (57.4%) and Euryarchaeota (22.3%) (Magnabosco et al., 2016). A second borehole at 3.14 km depth in the same mine was dominated by putative enzymes associated with the 3-hydroxypropionate/4-hydroxybutyrate cycle (Simkus et al., 2016). Further, the oxidation of alkanes is suspected to proceed by reversing the reductive acetyl-CoA pathway, and the abundance of related PEGs related may instead be used to oxidize alkanes (Magnabosco et al., 2016). The presence of methyl-coenzyme M reductase may signal methanogenesis among the Euryarchaea identified, but anaerobic oxidation of hydrocarbons (e.g., sulfate reduction coupled to propane or butane oxidation) yields more energy in these fluids than methanogenesis. It was also determined that the methane carries a depleted carbon isotopic signature consistent with a primarily abiotic origin at this site (Simkus et al., 2016). Carbon monoxide dehydrogenase was abundant in the community, along with the carboxydovore genus *Desulfotomaculum*, and CO oxidation coupled to H_2_O reduction was the second most energy yielding reaction considered (as energy flux, in units of kJ cell^–1^ s^–1^). These efforts expanded considerably what is known about carbon cycling in the deep terrestrial subsurface of the Witwatersrand Basin.

Finally, genes coding for enzymes involved in nitrogen cycling have been investigated in four Witwatersrand Basin mines. In all, seven nitrogen cycling genes (*Nar*V, NPD, *Nif*HDKEN) were found to be common to all samples analyzed (Lau et al., 2014). A further examination of the Beatrix fracture fluid utilized a combination of “omics” approaches to demonstrate that oxidation of sulfur species coupled to nitrate reduction by spp. of *Thiobacilus* and *Sulfuricella* accounted for 27.8% of the active microbial community (Lau et al., 2016). In addition, the key enzymes for dissimilatory nitrate reduction to ammonia, ANAMMOX, and nitrogen fixation were found in low quantities (Lau et al., 2016). However, isotopic evidence supports the conclusion that canonical denitrification is the primary nitrogen cycling process in this environment, with nitrate originating from either radiolytic oxidation or paleometeoric recharge (Silver et al., 2012). In order for *Thiobacilus* and *Sulfuricella* to drive this part of the nitrogen cycle, they require sufficient sulfur species to serve as electron donors. Lau et al. (2016) propose that these are produced by ANME-2 and sulfate reducing bacteria, which represent a smaller proportion of both the community and activity in this environment. In all, they describe an ecosystem that supports an “inverted” biomass pyramid, where the codependent methanogens and ANME members are active at low levels, and the sulfate reducing bacteria and methanogens are supported by the syntrophy with ANME and sulfur oxidizing bacteria. Carbon is moved through this system by autotrophy, primarily the reductive pentose phosphate cycle and reductive acetyl-CoA cycles, using DIC recycled through the methane cycling community. Contrary to previous assumptions supported by approaches that targeted hydrogen-driven metabolisms, this work showed that the bulk of the anabolic and catabolic activity in this 1.34 km fracture fluid did not use hydrogen as an electron donor, but rather hydrogen dependent metabolisms exist in syntrophy with coupled sulfur/nitrogen cycling (Lau et al., 2016), and called attention to the importance of the overlooked subsurface nitrogen cycle.

### Boreholes

Boreholes offer an indirect path to the subsurface and provide a means for emplacing instrumentation and long term experimentation *in situ*. Borehole depths are often comparable to deep mine investigations, frequently reaching 500–2,500 m. Dai et al. (2021) were able to retrieve continuous cores up to 5.1 km. As with other drilling operations interested in obtaining clean and uncontaminated samples for microbiological research, methodologies have been developed for terrestrial deep borehole drilling (e.g., Templeton et al., 2021). Here we consider some of the deepest examples of microbial surveys in boreholes in the continental crust, focusing on altered and metamorphosed bedrocks with lower permeability than sedimentary units, and thus less opportunity for migration of surface microorganisms (Mailloux et al., 2003; Lau et al., 2014). The examples below highlight boreholes into altered bedrocks, such as serpentinized ophiolites, schists, and gneisses, which offer fluid and gas chemistries that differ from the fracture fluids in the deep mine and laboratory examples above.

#### Boreholes in Serpentinized Bedrock

When ophiolites, interact with groundwater, the hydration of olivine and pyroxene within the rocks leads to the formation of a variety of serpentine phases such lizardite, chrysotile, among others (Moody, 1976, *and references therein;* Dilek and Furnes, 2011). These reactions lead to a production of H_2_, CH_4_, alkaline fluids, and a liberation of Ca^2+^ ions (e.g., Neal and Stanger, 1983; McCollom and Bach, 2009), providing the potential for the establishment of terrestrial chemoautotrophic microbiological communities that are largely divorced from inputs at the surface. Indeed, there are active microbial ecosystems in the deep subsurface fueled by serpentinization (reviewed previously in Schrenk et al., 2013). The origin of microorganisms within these subsurface communities is thought to be related to transportation to the subsurface during ophiolite obduction or introduction from groundwater along slow regional flow pathways (Sabuda et al., 2020; Putman et al., 2021). Microbial communities dependent on serpentinization are of particular interest to theories of the origin of life and potential life elsewhere in the solar system such as Mars or icy moons (e.g., Schulte et al., 2006; Martin et al., 2008; Cardace and Hoehler, 2009; Sojo et al., 2016; Preiner et al., 2018; Cartwright and Russell, 2019; Russell and Ponce, 2020; Taubner et al., 2020).

Well studied ophiolites include the Coast Range Ophiolite, United States, the Tablelands ophiolite, Canada, the Santa Elena ophiolite, Costa Rica, the Voltri Massif ophiolite, Italy, and the Samail ophiolite, Oman, among others. Until recently, much of the research on serpentinizing systems came from studies of alkaline rich springs, where serpentinization influenced groundwater intersects with the surface (Morrill et al., 2013; Crespo-Medina et al., 2014; Sánchez-Murillo et al., 2014; Cardace et al., 2015; Meyer-Dombard et al., 2015; Quéméneur et al., 2015; Woycheese et al., 2015; Brazelton et al., 2017; Canovas<suffix>III</suffix>, Hoehler and Shock, 2017; Rowe et al., 2017; Suzuki et al., 2017, 2018). Here, we focus on the direct sampling of fluids from borehole wells (see section “Surface Influences on the Terrestrial Subsurface”). In the last decade, drilling projects such as the Coast Range Ophiolite Microbial Observatory (CROMO) and the multi-borehole observatory (MBO) established by the Oman Drilling Project, have enabled more direct access to the groundwater fluids in contact with the subsurface (Cardace et al., 2013; Kelemen et al., 2013, Kelemen et al., 2020). Prior to the establishment of these observatories, direct access to groundwater of serpentinizing systems was rare (Tiago et al., 2004; Daae et al., 2013; Tiago and Veriìssimo, 2013).

Recent work utilizes next generation DNA sequencing for taxonomic and functional classification of borehole fluids in combination with other techniques such as microcosm or enrichment based experiments (Crespo-Medina et al., 2014; Meyer-Dombard et al., 2018; Fones et al., 2021; Glombitza et al., 2021), stable isotope analyses (Nothaft et al., 2021a,b), radioisotope labeling (Fones et al., 2021; Glombitza et al., 2021; Templeton et al., 2021), and ecological/hydrological modeling (Putman et al., 2021). Transcriptomic and single cell genomics studies have also shown mechanisms of adaptation of microbial life to serpentinizing environments (Fones et al., 2019, 2021; Merino et al., 2020; Kraus et al., 2021). The borehole observatories have enhanced the spatial and temporal resolution of microbial dynamics within serpentinizing systems and highlight the differences between borehole fluids influenced by the surface and deeper, highly reducing groundwater fluids.

Both host rock composition and hydrologic context influence microbial community composition in subsurface serpentinizing systems, and recent work at the MBO in Oman highlights this concept (Miller et al., 2016; Rempfert et al., 2017; Fones et al., 2019; Kraus et al., 2021; Nothaft et al., 2021a). The Samail Ophiolite is composed of mafic gabbros and ultramafic peridotites, mainly harzburgite, both presumed to be undergoing active serpentinization, which fuel distinct microbial communities (Neal and Stanger, 1983; Miller et al., 2016; Rempfert et al., 2017; Fones et al., 2019; Nothaft et al., 2021a). Rempfert et al. (2017) distinguished between four different fluid types at the Samail ophiolite MBO wells, influenced by host rock lithology and hydrologic flow regime (long v. short residence time): hyperalkaline peridotite (pH > 10), alkaline peridotite (pH 8–10), gabbro, and peridotite/gabbro contact wells. Taxonomic analysis showed microbial communities clustered in accordance with these four fluid types at the Samail Ophiolite (Rempfert et al., 2017). Geochemical data showed alkaline peridotite and gabbro hosted wells were likely to be more surface influenced, and harbored more nitrogen cycling microorganisms (Rempfert et al., 2017). Hyperalkaline peridotite wells, characterized by high dissolved H_2_, methane, and calcium, had more sulfate reducing Bacteria and methane cycling Archaea relative to other fluid types (Rempfert et al., 2017). Sixteen metagenome assemblies from the Samail MBO also showed metabolic potential correlated with fluid types associated with host rock lithologies (Fones et al., 2019). Metagenomes from hyperalkaline peridotite wells were more enriched in anaerobic respiration genes, including anaerobic sulfite reductase unit A, *AsrA*, while genes associated with aerobes were more enriched in the alkaline peridotite wells (< 10 pH) (Fones et al., 2019). The influence of hydrologic regime on geochemical composition, and thus microbial communities, was shown at the Samail Ophiolite through the use of packers to isolate discrete borehole sections in the mantle section of the ophiolite (Nothaft et al., 2021a). Nothaft et al. (2021a) sampled two boreholes at multiple discrete depths, up to 132 m, and subjected fluids to geochemical, isotopic, and 16s RNA sequencing. Fluids isolated from shallower, more oxidized parts of the groundwater contained more heterotrophic organisms capable of aerobic respiration, denitrification, and fermentation, while highly reacted Ca^2+^ – OH^–^ groundwaters were dominated by sulfate reducing chemolithoheterotrophs (Nothaft et al., 2021a).

Microbial communities in fluids most influenced by serpentinization are dominated by Bacteria, with variable archaeal abundances (< 1% to ∼30%), and generally consist of low richness and planktonic cell abundances (≤ 10^6^ cells mL^–1^) (e.g., Rempfert et al., 2017; Twing et al., 2017; Fones et al., 2019, 2021; Kraus et al., 2021; Putman et al., 2021; Sabuda et al., 2021). Studies associated with rock solids in these environments are uncommon, but microscopy and 16S rRNA sequencing from cores obtained during drilling at CROMO and at MBO indicated variable cell abundances (10^3^–10^7^ cells per gram) and low taxonomic richness (Cardace et al., 2013; Kelemen et al., 2020; Templeton et al., 2021). Selective pressures that affect community structure in these environments include low dissolved inorganic carbon (DIC) concentrations, high pH, low dissolved oxygen (DO), and energy limitations (Rempfert et al., 2017; Twing et al., 2017; Fones et al., 2019, 2021; Nothaft et al., 2021a; Putman et al., 2021). While microbial populations in highly reducing, hyperalkaline groundwater are generally limited by the lack of more energy rich electron acceptors (oxidants such as O_2_, NO_3_^–^, and Mn; e.g., Twing et al., 2017), this may not be the most important limitation on microbial metabolisms (Crespo-Medina et al., 2014; Rempfert et al., 2017; Glombitza et al., 2021). Enrichment experiments show low DIC may be most limiting, driving the selection for unique adaptations such as higher rates of substrate assimilation to dissimilation, and methanogen diversification (Crespo-Medina et al., 2014; Fones et al., 2019, 2021; Kraus et al., 2021). Finally, ecological modeling has shown dispersal limitation due to low permeability and slow fluid flow imposes strong selective pressure on microbial communities within ophiolite hosted aquifers at CROMO (Putman et al., 2021), leading to microbial community differentiation.

As highlighted above, the functional potential of serpentinizing communities depends on the availability of substrates such as H_2_ and CH_4_, which can be highly variable depending on the depth, host rock lithology, and the extent of surface influence (Daae et al., 2013; Rempfert et al., 2017; Nothaft et al., 2021a; Sabuda et al., 2021). Some ophiolite hosted aquifers may retain ancient seawater from their marine origins, which contributes to increased salinity and dissolved sulfate in some sites, potentially providing a resource that could be utilized by microbial communities (Schwarzenbach et al., 2012; Sabuda et al., 2020; Nothaft et al., 2021a; Putman et al., 2021). Active microbially mediated sulfur cycle processes are abundant in aquifers with higher sulfate concentrations (Schrenk et al., 2013; Sabuda et al., 2020; Glombitza et al., 2021). Metagenomic sequencing has detected genes coding for the complete dissimilatory sulfate reduction pathway such as sulfate adenylytransferase (*sat*), adenosine-5′-phosphosulfate reductase (*aprAB*) and dissimilatory sulfite reductase (*dsrAB*) (Sabuda et al., 2020; Glombitza et al., 2021). Low levels of sulfate reduction comparable with those found in deep subseafloor sediments, < 10^–3^ to 2.1 pmol mL^–1^day^–1^, were detectable up to pH 12.3 in laboratory incubations of serpentine fluids isolated from both CROMO and MBO wells (Glombitza et al., 2021). Incubations of 10 cm core samples at pH 9.5 from MBO yielded sulfate reduction rates of 2–1,000 fmol cm^–3^day^–1^ (Templeton et al., 2021). At the CRO, sulfate reduction coupled to methane oxidation was found to be most thermodynamically favorable in the deeper wells, as well as sulfide and thiosulfate oxidation (Sabuda et al., 2020). Sulfate reducing bacteria (SRB) *Thermodesulfovibrionaceae* and *Desulforudaceae* are commonly found at both Coast Range and Samail ophiolite fluids (Sabuda et al., 2020; Glombitza et al., 2021; Nothaft et al., 2021a; Templeton et al., 2021). Glombitza et al. (2021) found that SRB constituted 39.59% of taxa in the Samail and Coast Range Ophiolites by screening taxonomic ranks for possession of *dsrAB* genes. It has been suggested that ophiolites serve as a vector for transporting sulfur compounds and microorganisms capable of sulfur metabolism from the seafloor to the continents, highlighting sulfur biogeochemistry taking place within ophiolites as a link between terrestrial and marine systems (Sabuda et al., 2020).

Metagenomic and 16S rRNA gene sequencing evidence for hydrogen metabolisms, nitrogen cycling metabolisms, carbon monoxide oxidation, carbon fixation, acetogenesis, sulfate reduction, sulfide oxidation, and to a lesser extent, methane oxidation have been found in serpentinizing boreholes *in situ* (e.g., Twing et al., 2017; Fones et al., 2019; Merino et al., 2020; Kraus et al., 2021; Sabuda et al., 2021). Ferrous iron has been measured in borehole samples, indicating the potential for iron redox metabolisms (Miller et al., 2016; Rempfert et al., 2017). Several methods have shown that microbially mediated iron reduction and oxidation occur in enrichment microcosms and batch culture experiments using fluids from subsurface borehole and hyperalkaline springs (Meyer-Dombard et al., 2018). While iron reduction is thought to be unfavorable in hyperalkaline conditions, local drops in pH (< 9 pH) driven by fractures, low flow voids, or biofilm protection can potentially create favorable conditions. Borehole measurements show that pH in the Samail and Coast Range ophiolites can range between ∼ 7.4 to > 12 pH (Rempfert et al., 2017; Putman et al., 2021), potentially providing the context necessary for iron oxidation at the lower end of this range.

Methanogenesis is often cited as an energetically favorable potential metabolism in serpentinizing fluids, however, the observation of isotopically heavy methane in the “abiotic” synthesis range has garnered some debate on the origin of methane in these systems (Etiope et al., 2016; Miller et al., 2016, Canovas<suffix>III</suffix>, Hoehler and Shock, 2017; Etiope, 2017). It is likely that a combination of abiotic and biogenic methane is present in serpentinizing systems, as genetic evidence for methanogens within borehole fluids has been reported (Miller et al., 2016; Fones et al., 2021; Kraus et al., 2021; Nothaft et al., 2021b). Methanogenesis under DIC limitation has been shown to result in relatively high δ^13^C-CH_4_ values, potentially providing a mechanistic explanation for the observed isotopically heavy methane (Miller et al., 2018). Methanotrophy may provide another mechanism for δ^13^C enrichment (Nothaft et al., 2021b). It has been suggested that biological methanogenesis becomes energetically competitive with sulfate reduction at a threshold level of accumulation of reduced compounds such as H_2_ or formate in the range of 10–10^2^ μmol per L in the Samail ophiolite, which is a higher threshold than other environments potentially due to increased energy expenditure to cope with high pH (Nothaft et al., 2021a). Several studies have identified methanogens affiliated with *Methanobacterium*, an autotrophic and hydrogenotrophic genus (Rempfert et al., 2017; Kraus et al., 2021; Nothaft et al., 2021a). Genomic and transcriptomic evidence suggest that *Methanobacterium* can be active in hyperalkaline groundwater up to pH 11.3 (Kraus et al., 2021). Taxonomic evidence for aerobic and anaerobic methane oxidation is available to lesser extent than methanogenesis, however, both are predicted to be energetically favorable in these environments (Miller et al., 2016; Canovas<suffix>III</suffix>, Hoehler and Shock, 2017; Twing et al., 2017; Nothaft et al., 2021b; Sabuda et al., 2021). Methane produced either biogenically or abiotically may not be consumed to a significant degree by methanotrophs, and methane emissions from sites of serpentinization should be considered in global atmospheric methane budgets (Sabuda et al., 2021).

#### Boreholes in the Fennoscandian Shield

Boreholes fluids at the Outokumpu site (Finland) in the Fennoscandian Shield have been used extensively as an *in situ* experimental canvas. Here, the lithology of the first 1.3 km is organic rich mica schists below which continues an ophiolite, ultramafic sequence (Nyyssönen et al., 2014), providing a comparison with the serpentinized fluids discussed above. Emphasis has been placed on down hole experimentation, as well as use of inflatable packer systems to retrieve authentic deep subsurface fluid samples. Early work identified sulfate reduction in fracture fluids (*via* identification and enumeration of the *dsrB* gene) at varying depths (Itävaara et al., 2011; Purkamo et al., 2013). However, the organic rich schists of the Outokumpu site provides opportunity for fermentation and anaerobic respiration, and initial predictions of functional diversity indicated that carbon fixation processes were underrepresented in the microbial communities (Purkamo et al., 2015a,b), although the genetic capacity for the reductive acetyl-CoA pathway is found in fluids from 600–2,300 m (Nyyssönen et al., 2014). Enrichments with acetate, sulfate, H_2_, and CO_2_ in laboratory microcosms clarified that acetate induced growth of bacterial heterotrophs, thiosulfate reduction was more important that sulfate reduction, and methanogens were not present (Purkamo et al., 2017). However, a recent metagenomic survey of fluids at 600, 1,500, and 2,300 m detected the genetic capacity for methanogenesis from multiple pathways; acetoclastic and methylotrophic methanogenesis pathways were found at all depths, while hydrogenotrophic pathways were only found in the deepest sample (Nyyssönen et al., 2014). Further work demonstrated that acetate was incorporated into biomass by archaeal members of the community, supporting previous indications that acetate is likely key for metabolism in the deep biosphere at 2.2 km at Outokumpu (Nuppunen-Puputti et al., 2018).

A variety of other locations provide fracture fluids within metamorphic units in the Fennoscandian Shield. Shallow boreholes provide access to Precambrian gneisses (e.g., at Olkiluoto, Romuvarra), where emphasis on cultivation and identification of sulfate reducing bacteria, nitrate reducing bacteria, acetogens, and methanogens has been the focus of much previous research (Nyyssönen et al., 2012; Pedersen, 2013; Bomberg et al., 2015, 2016; Kutvonen et al., 2015; Miettinen et al., 2018). For example, in shallow fluids (up to 600 m), sulfate reduction and methanotrophy were induced when hydrogen and methane were introduced in flow cells using fluid from a borehole in the ONKALO tunnel (Olkiluoto, Finland; Pedersen, 2013), and active nitrate and ammonium based catabolism were also indicated (Kutvonen et al., 2015; Miettinen et al., 2018). In contrast to many other Fennoscandian Shield sites, at Romuvaara, groundwaters are not saline, and Purkamo et al. (2018) postulated that this was the reason for a different suite of microbial community compositions, despite similar host rock as Olkiluoto. Interestingly, Romuvaara groundwaters from ∼600 m depth demonstrated a similar suite of predicted functional diversity as that found in Olkiluoto. One study investigated metabolic functions at much greater depth; a deep borehole (4.5 km) into gneiss bedrock was obtained at the Otaniemi site in Finland. Here, the functional diversity of retrieved rock (as opposed to groundwater) was determined *via* qPCR of *drsB*, *narG*, and *mcrA* genes (Purkamo et al., 2020). This work was only able to retrieve copies of the *narG* gene from this deeper borehole site, potentially suggesting that nitrate reduction outcompetes sulfate reduction and methanogenesis at depths greater than 600 m in gneissic bedrock. Other potential metabolic processes were predicted from 16S rRNA data, and indicated that a variety of chemoheterotrophic and methylotrophic metabolic options could also be present at these depths.

It is tempting to compare the functional diversity of the schist-hosted vs. gneiss-hosted deep biosphere in the Fennoscandian Shield, at depths > 1 km. It could be said that the schist hosted deep biosphere appears to support a more acetate driven ecosystem, with indications that thiosulfate reduction is also important, and that nitrogen cycling may be more important in the gneiss hosted deep biosphere. However, caution should be taken in these broad conclusions as different specific metabolic functions were the target of the searches in each location. For example, while experimental approaches have shown that ^15^NH_4_^+^ and ^15^NO_3_^–^ is taken up into biomass at Olkiluoto (100 m; Kutvonen et al., 2015), similar experiments have not yet been applied to verify the activity of the nitrogen cycling genes at greater depths at this site (Purkamo et al., 2020). Metagenomic data suggests that nitrogen cycling may be important in the schist/serpentine hosted Outokumpu groundwaters, but data showing activity of these genes are not yet available. Future analyses that broadly compare functional capacity and show activity of the genes involved or uptake into biomass will enhance our understanding of metabolic regimes across host rock types and geochemical profiles.

## Synthesis *via* Catabolic Modeling

Many have predicted the energetic landscape that might be presented in the terrestrial subsurface (e.g., Stevens and McKinley, 1995; Amend and Teske, 2005; Osburn et al., 2014; Magnabosco et al., 2016; Canovas<suffix>III</suffix>, Hoehler and Shock, 2017). Because the interplay between climate, topography, host rock, and groundwater defines the terrestrial subsurface environment, it has been suggested that, other environmental parameters being equal, the energetic landscape of the subsurface biosphere is dependent on the mineralogy of the host rock. Specifically, the host rock determines the mineralogy of the substrates that then serve as the backbone of the energetic “buffet” for chemosynthetic organisms (e.g., Swanner and Templeton, 2011; Casar et al., 2021a,b). Further, groundwaters connect diverse rock types, adding the history of the water rock interaction along the flow path with the local host rock, producing a distinctive fracture fluid chemistry (e.g., Sahl et al., 2008; Osburn et al., 2019).

This geochemical reality provides a stunning array of possibilities to supply the subsurface with energy and carbon. On the whole, the community has generally and historically made the assumptions that, (1) when methane or hydrogen gas are measurable in subsurface fluids, the biosphere is likely dominated by sulfate reduction and methanogenesis, and (2) presence of heterotrophic metabolisms indicates influence from the surface biosphere. These assumptions made a great deal of sense several decades ago, but have been consistently shown to be less relevant in many locations as more data are acquired, reduced sulfur species, ammonium, ferrous iron and other metals are shown to be important sources of electrons, and abiotic reactions resulting in synthesis of organic carbon are known to provide fuel for biomass synthesis (Amend and Teske, 2005; McCollom et al., 2010; Shock and Canovas, 2010; Osburn et al., 2014; Bomberg et al., 2019). Very recent considerations of the structure of the energetic landscape in deep continental biosphere settings have begun to paint a picture that methanogenesis may be limited at greater depths, or more prevalent where fresh groundwater mixes with deeper fluids (e.g., Osburn et al., 2014; Leong and Shock, 2020). Holistic and comprehensive analysis of functional diversity, capacity, and profiling of energy availability using updated databases and culture independent methods are suggesting that, where methanogens or the genetic capacity for methanogenesis are found in low levels in fracture fluids (and are not necessarily universally found, e.g., Lau et al., 2014), methanogenesis may not be the dominant metabolic process but rather may be providing a support for other organisms (Lau et al., 2016; Purkamo et al., 2016; Simkus et al., 2016). Additional ecological functions such as nitrogen cycling, methane oxidation, and iron cycling have emerged as metabolisms of interest in discussions of terrestrial deep subsurface sites (Swanner and Templeton, 2011; Lau et al., 2014, 2016; Magnabosco et al., 2016; Momper et al., 2017b; Purkamo et al., 2020; Casar et al., 2021b).

**FIGURE 2:**
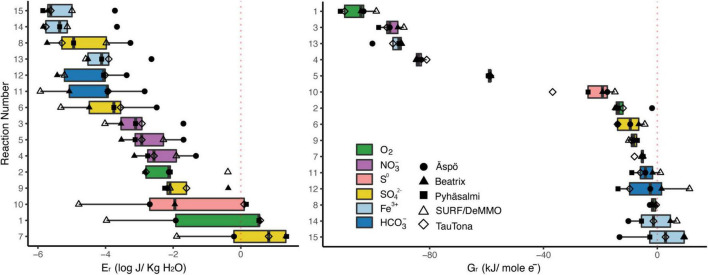
Estimated energetic landscape of the fifteen reactions presented in Table 1 for five selected deep fracture fluids, as accessed in subsurface laboratories. Calculated energy densities are presented at left, and ΔG_*r*_ estimates are at right (normalized as kJ/mol and the number of electrons involved in the reaction). Electron acceptors for each reaction are highlighted. Fracture fluid geochemistry data are from: Aspö - Hallbeck and Pedersen (2008), sample “KJ0052F01/2006-03-23/43.70-43.90 m;” Beatrix mine – Lin et al. (2006b), sample “BE325;” Pyhäsalmi – Miettinen et al. (2015), sample “R-2247;” SURF – Osburn et al. (2014), sample “‘Manifold B;” TauTona – Magnabosco et al. (2016), sample “TT107.” Methods for estimated values may be found in Supplementary Material.

To better ascertain whether there is a universality to energy availability in terrestrial subsurface sites, we attempted to canvas the known literature for published values to compare sites. The representation of “energy” is not standardized, and available data are presented in a dazzling variety of metrics and normalizations. Data are often presented only in figures, preventing the conversion of published data to a consistent metric across sites. Further complicating the process is variable reporting of geochemical data. For example, aqueous chemistry is frequently presented without corresponding gas data, measurements of redox sensitive compounds (such as NH_4_^+^, HS^–^), or fluid conductivity, which are essential for calculation of ΔG_*r*_. We have used geochemical data where available to estimate energy availability and density for deep subsurface fracture waters across five different deep mine laboratory locations – Äspö, Beatrix, Pyhäsalmi, SURF/MeMMO, and TauTona. We chose fifteen reactions of interest to further our discussion of the energetic landscape for these systems, shown in Table 1. The results of our estimates are found in Figure 2 and in Supplementary Table 1, where we present the data as both “energy density” and in the semi standard metric of ΔG_*r*_, normalized per mole of electrons involved in each reaction. Convincing arguments have been made for these metrics and normalizations, which present the data as volumetric and molar distributions of energy, respectively (McCollom, 2000; Amend and Shock, 2001; LaRowe and Amend, 2014, 2019).

We include reactions that are commonly exergonic in terrestrial deep biosphere systems (e.g., reactions 1, 6, 11), and that include nitrate and oxidized iron minerals as an electron acceptor (processes currently of interest). We also chose reactions that specifically do not involve hydrogen as an electron donor to compare to reactions that do (e.g., reactions 3 vs. 4, 6 vs. 9). We represent sulfate reduction with both hydrogen and methane, but also acetate, and chose to reduce HCO_3_^–^ to methane because that is the dominant species at the pH ranges of the systems in question. Further, in Figure 2 we highlight the electron acceptors involved in each reaction, as fluids at all examined sites are reducing and these are the chemical species receiving the transferred electrons.

In general, on a molar basis, the most exergonic reactions across all sites are those using electron acceptors in the order O_2_ > NO_3_^–^ > S^0^ > SO_4_^–2^ > HCO_3_^–^ > Fe-minerals (Figure 2, right side). Under this lens, there is little variability in the energy available per reaction across the five locations, with most reactions varying < 10 kJ(mol e^–^)^–1^. Of interest are the reactions that play across the zero line, showing that the reaction is only (barely) exergonic at some sites. These are methanogenesis, acetogenesis, sulfate reduction with methane, and reduction of iron bearing minerals with H_2_ (reactions 11, 12, 8, 14, and 15, respectively). These same reactions also provide the least energy on a volumetric basis, with Aspö fluids providing the most energy of the five sites (Figure 2, right side). The “top 5” exergonic reactions differ considerably when assessed as volumetric vs. molar normalizations. Normalized per a volumetric basis, the most exergonic reactions across all sites are 7, 1, 10, 9, and 2, which include sulfate reduction with methane or acetate (7, 9), sulfur reduction with H_2_ (10), and ferrous iron oxidation (2). Normalized per a molar basis, only one of these reactions remains in the “top 5” - reaction 1 - joined by nitrate reduction with H_2_, HS^–^, and CH_4_, and ferrihydrite reduction coupled with acetate (reactions 3–5, and 13, respectively). Note that there is considerable variability in the energy density for the “top 5” reactions among sites, with the DeMMO fracture fluids presenting far less energy density for the top 3 reactions than all the other sites (reaction 2 is actually the most energy dense for DeMMO fluids).

We can choose thresholds by which to compare the sites’ energy density. On a site by site comparison, there are seven reactions for which the Aspö fluids yield > −2 (log J/kg H_2_O) and only 4–5 reactions in this range for all the other sites. If we look at the lower energy density threshold of < −4 (log J/kg H_2_O), there are ten reactions for which the DeMMO fluid yields this or less energy on a volumetric basis, and none of the Aspö reactions fall in this range. Ranking the sites in this way, the Aspö site had the greatest number of high energy density reactions, the DeMMO fluids had the least, and Beatrix, TauTona, and Pyhäsalmi were midrange, in descending order. Fracture fluids at Aspö are flowing through basaltic units, while Beatrix and TauTona are hosted in quartzites. Both Pyhäsalmi and DeMMO fluids experience more complex geologic settings and reaction pathways. One might conclude that there is a pattern here, from the most energy dense site to the least, with sites hosted in similar geologic settings arranged in a gradient, but more data would be needed to verify this. Pan-metagenomic analysis of global subsurface sites suggests that community variations are correlated to lithology, so the possibility exists that this trend would hold up to deeper scrutiny (Magnabosco et al., 2018).

Looking at specific reactions of interest, we can compare the reduction of nitrate and sulfate with and without hydrogen, toward considering whether the subsurface might be dependent on hydrogen as an electron donor. Reactions 3 and 4 (nitrate reduction with hydrogen and with sulfide, respectively) yield roughly the same energy density for nearly all sites, although DeMMO fluids are significantly more energy dense when sulfide is the electron donor. Reactions 6–9 all reduce sulfate; of these, reducing sulfate with hydrogen is one of the least energy dense options. Reduction of sulfate to sulfide with methane is the most energy dense of all the reactions, but reducing sulfate to elemental sulfur with methane yields considerably less energy density. Sulfate reduction with methane, acetate, and hydrogen are all more energy dense than methanogenesis, for each site. Stacking the four yellow bars in the left plot of Figure 2 covers the entire range of energy density shown on the plot, when all samples are included. This indicates that sulfate reduction is highly site and reaction specific, which should be taken into consideration when discussing this as a metabolic option in the subsurface. Nitrate reduction is a metabolism that has been highlighted above in several deep subsurface mine publications, and notably, reactions 3–5 are among the most exergonic when considered on a molar basis and are also in the middle range of the scale when considered on a volumetric basis. Further, across all sites there is less range in the energy density available for nitrate reduction, indicating that it is less site and reaction specific. We believe that reactions utilizing nitrate as an electron acceptor warrant closer inspection and consideration in metabolic profiling for subsurface fracture fluids, especially given recent discoveries concerning the genetic capacity for these processes (Swanner and Templeton, 2011; Lau et al., 2014, 2016; Magnabosco et al., 2016; Momper et al., 2017b; Purkamo et al., 2020). Whether methane oxidation (reaction 5, 7, and 8) is an energy dense option depended on what is being used as the electron acceptor, and those data overlap to cover the entire x-axis range in Figure 2. Again, this indicates that methane oxidation as an energy dense option depends highly on site chemistry and reaction specifics. Magnabosco et al. (2018) isotopically traced methane oxidation with nitrate in fracture fluids in the Beatrix mine, and our estimates indicate that, at least with reaction 5, there is a high energy density present for this process. Finally, we considered whether iron cycling is a favorable process in these fluids. This is site dependent. For the most part, reduction of iron bearing minerals is not a highly exergonic option, considering both volumetric and molar values of energy. However, reaction 13 yielded as much energy in the Aspö fluids as ferrous iron oxidation (reaction 2) on a volumetric basis and the reaction is highly exergonic on a molar basis. Ferrous iron oxidation with oxygen is one of the “top 5” reactions for all sites on a volumetric basis. Iron cycling may be a possible source of energy in subsurface fracture fluids, but generalizations can’t be drawn across sites.

We recognize that the presence of energy availability does not confer the guarantee of the importance of a given process to an ecosystem, any more than identification of taxonomic or functional diversity might. While the choice of how metrics of energy availability and flux are normalized depends largely on the questions being asked in a given study, it is certain that more cross-site comparisons could be made if, at the very least, the ΔG_*r*_ value is reported along with the normalized data as a discipline standard. Further, some basic standards could be suggested for data collection that would enable others to assess the energy availability of a system, even if the study’s authors are disinterested in that topic. For example, analysis of temperature, pH and conductivity, as well as redox pairs (nitrate and ammonia/ammonium, sulfate and sulfide, for example) and gas chemistry that includes dissolved oxygen, hydrogen, nitrogen, and methane, could serve as a general “basic set” of useful analyses (with acknowledgment that analytical capacity varies widely in the field). The most convincing investigations of ecosystem function combine modeling of energy options, functional diversity surveys, and experimental approaches. We look forward to the community putting the “nail in the coffin” by adding proteomic analysis to such suites of data, which will greatly enhance our ability to draw connections between geology, geochemistry, and community diversity to describe ecosystem functions and services in the deep terrestrial subsurface biosphere.

## Conclusion

The mass of the terrestrial subsurface biosphere has been frequently estimated, with current values conservatively approaching 23–31 Pg of carbon C (PgC) (Magnabosco et al., 2018). There are many environmental factors that are very similar in most terrestrial subsurface sites, and several studies have considered taxonomic and functional diversity from a global perspective. Several taxa have been noted in many subsurface sites globally, regardless of geochemical provenance (Colman et al., 2017). Among these are members of the Firmicutes and Proteobacteria. Firmicutes are shown to dominate taxonomic diversity surveys in deeper subsurface sites in South Africa (Magnabosco et al., 2016), various serpentinite sites including surface springs (Brazelton et al., 2013; Suzuki et al., 2013; Twing et al., 2017), and fracture fluids in boreholes and deep laboratories of the Fennoscandian Shield (Itävaara et al., 2011; Purkamo et al., 2016), while Proteobacteria dominate in shallower sites that mix with younger and less saline groundwaters (Itävaara et al., 2011; Magnabosco et al., 2016). The well-noted *Candidatus Desulforudis audaxviator*, a Firmicutes, has been found in subsurface fluids globally since first discovered in a South African gold mine (Baker et al., 2003; Cowen et al., 2003; Lin et al., 2006a,b; Aüllo et al., 2013; Tiago and Veriìssimo, 2013; Labonteì et al., 2015; Magnabosco et al., 2016; Jungbluth et al., 2017; Momper et al., 2017b). Notably, fluids across these sites are geochemically diverse, and the presence of the same taxonomic groups suggests both functional redundancy and functional generalists within the communities. Indeed, it was shown that distributions of functional genes are not correlated across sites, geochemistry, distance, or other physical/environmental parameters (Lau et al., 2014).

As we move forward in terrestrial subsurface research, it will be important to consider subjects such as functional redundancy and the ecosystems services that both generalist and specialist taxa provide. Narrowly targeting investigations to a few specific metabolic functions can help elucidate details, but holistic context should not be neglected. To facilitate such progress, we look forward to coordinated analyses that examine the energetic landscapes, taxonomic and functional diversity, and end point analyses such as proteomics and enzymatic assays.

## Author Contributions

Both authors listed have made a substantial, direct, and intellectual contribution to the work, and approved it for publication.

## Conflict of Interest

The authors declare that the research was conducted in the absence of any commercial or financial relationships that could be construed as a potential conflict of interest.

## Publisher’s Note

All claims expressed in this article are solely those of the authors and do not necessarily represent those of their affiliated organizations, or those of the publisher, the editors and the reviewers. Any product that may be evaluated in this article, or claim that may be made by its manufacturer, is not guaranteed or endorsed by the publisher.

## References

[B1] Amend J. P. Shock E. L. (2001). Energetics of overall metabolic reactions of thermophilic and hyperthermophilic Archaea and Bacteria. *FEMS Microbiol. Rev.* 25 175–243. 10.1111/j.1574-6976.2001.tb00576.x 11250035

[B2] Amend J. P. Teske A. (2005). Expanding frontiers in deep subsurface microbiology. *Paleogeography Paleoclimatol. Palaeoecol.* 219 131–155. 10.1016/b978-0-444-52019-7.50012-7

[B3] Anantharman K. Brown C. T. Hug L. A. Sharon I. Castelle C. J. Probst A. J. (2016). Thousands of microbial genomes shed light on interconnected biogeochemical processes in an aquifer system. *Nat. Commun.* 7:13219. 10.1038/ncomms13219 PMC5079060 27774985

[B4] Aüllo T. Ranchou-Peyruse A. Ollivier B. Magot M. (2013). *Desulfotomaculum spp*. and related gram-positive sulfate-reducing bacteria in deep subsurface environments. *Front. Microbiol.* 4:362. 10.3389/fmicb.2013.00362 24348471 PMC3844878

[B5] Bagnoud A. Chourey K. Hettich R. L. de Bruijn I. Andersson A. F. Leupin O. X. (2016). Reconstructing a hydrogen-driven microbial metabolic network in Opalinus Clay rock. *Nat. Commun.* 7:12770. 10.1038/ncomms12770 27739431 PMC5067608

[B6] Baker B. J. Moser D. P. MacGregor B. J. Fishbain S. Wagner M. Fry N. K. (2003). Related assemblages of sulphate-reducing bacteria associated with ultradeep gold mines of South Africa and deep basalt aquifers of Washington State. *Environ. Microbiol.* 5 267–277. 10.1046/j.1462-2920.2003.00408.x 12662174

[B7] Barton H. A. Northup D. E. (2007). Geomicrobiology in cave environments: past, current and future perspectives. *J. Cave Karst Stud.* 69 163–178.

[B8] Bomberg M. Lamminmäki T. Itävaara M. (2016). Microbial communities and their predicted metabolic characteristics in deep fracture groundwaters of the crystalling bedrock at Olkiluoto, Finland. *Biogeosciences* 13 6031–6047. 10.5194/bg-13-6031-2016

[B9] Bomberg M. Mäkinen J. Salo M. Kinnunen P. (2019). High diversity in iron cycling microbial communities in acidic, iron-rich water of the pyhäsalmi mine, Finland. *Geofluids* 2019:7401304. 10.1155/2019/7401304

[B10] Bomberg M. Nyyssönen M. Pitkänen P. Lehtinen A. Itävaara M. (2015). Active microbial communities inhabit sulphate-methane interphase in deep bedrock fracture fluids in Olkiluoto, Finland. *BioMed Res. Int.* 2015:979530. 10.1155/2015/979530 26425566 PMC4573625

[B11] Boyd E. S. Cummings D. E. Geesey G. G. (2007). Mineralogy influences structure and diversity of bacterial communities associated with geological substrata in a pristine aquifer. *Microb. Ecol.* 54 170–182. 10.1007/s00248-006-9187-9 17364247

[B12] Brazelton W. J. Morrill P. L. Szponar N. Schrenk M. O. (2013). Bacterial communities associated with subsurface geochemical processes in continental serpentinite springs. *Appl. Environ. Microbiol.* 79 3906–3916. 10.1128/AEM.00330-13 23584766 PMC3697581

[B13] Brazelton W. J. Thornton C. N. Hyer A. Twing K. I. Longino A. A. Lang S. Q. (2017). Metagenomic identification of active methanogens and methanotrophs in serpentinite springs of the Voltri Massif, Italy. *PeerJ* 5:e2945. 10.7717/peerj.2945 28149702 PMC5274519

[B14] Canovas P. C. III Hoehler T. Shock E. L. (2017). Geochemical bioenergetics during low-temperature serpentinization: an example from the Samail ophiolite, Sultanate of Oman. *J. Geophys. Res. Biogeosci.* 122 1821–1847. 10.1002/2017JG003825

[B15] Cardace D. Hoehler T. M. (2009). Serpentinizing fluids craft microbial habitat. *Northeastern Nat.* 16 272–284. 10.1656/045.016.0520 22708719

[B16] Cardace D. Hoehler T. McCollom T. Schrenk M. Carnevale D. Kubo M. (2013). Establishment of the Coast Range ophiolite microbial observatory (CROMO): drilling objectives and preliminary outcomes. *Sci. Drilling* 16 45–55. 10.5194/sd-16-45-2013

[B17] Cardace D. Meyer-Dombard D. R. Woycheese K. M. Arcilla C. A. (2015). Feasible metabolisms in high pH springs of the Philippines. *Front. Microbiol.* 6:10. 10.3389/fmicb.2015.00010 25713561 PMC4322734

[B18] Cartwright J. H. E. Russell M. J. (2019). The origin of life: the submarine alkaline vent theory at 30. *Interface Focus* 9:20190104. 10.1098/rsfs.2019.0104

[B19] Casar C. P. Kruger B. R. Flynn T. M. Osburn M. R. (2020). Mineral-hosted biofilm communities in the continental deep subsurface, Deep Mine Microbial Observatory, SD, USA. *Geobiology* 18 508–522. 10.1111/gbi.12391 32216092

[B20] Casar C. P. Kruger B. R. Osburn M. R. (2021a). Rock-Hosted subsurface biofilms: mineral selectivity drives hotspots for intraterrestrial life. *Front. Microbiol.* 12:658988. 10.3389/fmicb.2021.658988 33897673 PMC8062869

[B21] Casar C. P. Momper L. M. Kruger B. R. Osburn M. R. (2021b). Iron-Fueled life in the continental subsurface: deep mine microbial observatory, South Dakota, USA. *Appl. Environ. Microbiol.* 87:e00832-21. 10.1128/AEM.00832-21 34378953 PMC8478452

[B22] Chivian D. Brodie E. L. Alm E. J. Culley D. E. Dehal P. S. DeSantis T. Z. (2008). Environmental genomics reveals a single-species ecosystem deep within Earth. *Science* 322 275–278. 10.1126/science.1155495 18845759

[B23] Christner B. C. Priscu J. C. Achberger A. M. Barbante C. Carter S. P. Christianson K. (2014). A microbial ecosystem beneath the West Antarctic ice sheet. *Nature* 512 310–313. 10.1038/nature13667 25143114

[B24] Cockell C. S. Holt J. Campbell J. Groseman H. Josset J.-L. Bontognali T. R. R. (2019). Subsurface scientific exploration of extraterrestrial environments (MINAR 5): analog science, technology and education in the Boulby Mine. UK. *Int. J. Astrobiol.* 18 157–182. 10.1017/s1473550418000186

[B25] Colman D. R. Feyhl-Buska J. Fecteau K. M. Xu H. Shock E. L. Boyd E. S. (2016). Ecological differentiation in planktonic and sediment-associated chemotrophic microbial populations in Yellowstone hot springs. *FEMS Microbiol. Ecol.* 92:fiw137. 10.1093/femsec/fiw137 27306555

[B26] Colman D. R. Poudel S. Stamps B. W. Boyd E. S. Spear J. R. (2017). The deep, hot biosphere: twenty-five years of retrospection. *Proc. Natl. Acad. Sci. U S A.* 113 6895–6903. 10.1073/pnas.1701266114 28674200 PMC5502609

[B27] Cowen J. P. Giovannoni S. J. Kenig F. Johnson H. P. Butterfield D. Rappeì M. S. (2003). Fluids from aging ocean crust that support microbial life. *Science* 299 120–123. 10.1126/science.1075653 12511653

[B28] Crespo-Medina M. Twing K. I. Kubo M. D. Hoehler T. M. Cardace D. McCollom T. (2014). Insights into environmental controls on microbial communities in a continental serpentinite aquifer using a microcosm-based approach. *Front. Microbiol.* 5:604. 10.3389/fmicb.2014.00604 25452748 PMC4231944

[B29] Daae F. L. Okland I. Dahle H. Jorgensen S. L. Thorseth I. H. Pedersen R. B. (2013). Microbial life associated with low-temperature alteration of ultramafic rocks in the Leka ophiolite complex. *Geobiology* 11 318–339. 10.1111/gbi.12035 23551703

[B30] Dai X. Wang Y. Luo L. Pfiffner S. M. Dong Z. Xu Z. (2021). Detection of the deep biosphere in metamorphic rocks from the Chinese continental scientific drilling. *Geobiology* 19 278–291. 10.1111/gbi.12430 33559972

[B31] D’Angeli I. M. Ghezzi D. Leuko S. Firrincieli A. Parise M. Fiorucci A. (2019). Geomicrobiology of a seawater-influenced active sulfuric acid cave. *PLoS One* 14:e0220706. 10.1371/journal.pone.0220706 31393920 PMC6687129

[B32] Davidson M. M. Silver B. J. Onstott T. C. Moser D. P. Gihring T. M. Pratt L. M. (2011). Capture of planktonic microbial diversity in fractures by long-term monitoring of flowing boreholes, evander basin, South Africa. *Geomicrobiol. J.* 28 275–300. 10.1080/01490451.2010.499928

[B33] De Waele J. Audra P. Madonia G. Vattano M. Plan L. D’Angeli I. M. (2016). Sulfuric acid speleogenesis (SAS) close to the water table: examples from southern France, Austria, and Sicily. *Geomorphology* 253 452–467. 10.1016/j.geomorph.2015.10.019

[B34] DeLeon-Rodriguez N. Lathem T. L. Rodriguez-R L. M. Barazesh J. M. Anderson B. E. Beyersdorf A. J. (2013). Microbiome of the upper troposphere: species composition and prevalence, effects of tropical storms, and atmospheric implications. *Proc. Natl. Acad. Sci. U S A.* 110 2575–2580. 10.1073/pnas.1212089110 23359712 PMC3574924

[B35] Dong Y. Gupta Kumar C. Chia N. Kim P.-J. Miller P. A. Price N. D. (2014). Halomonas sulfidaeris-dominated microbial community inhabits a 1.8 km-deep subsurface Cambrian Sandstone reservoir. *Environ. Microbiol.* 16 1695–1708. 10.1111/1462-2920.12325 24238218

[B36] Dilek Y. Furnes H. (2011). Ophiolite genesis and global tectonics: geochemical and tectonic fingerprinting of ancient oceanic lithosphere. *Geol. Soc. Am. Bullet.* 123, 387–411.

[B37] Ekendahl S. Pedersen K. (1994). Carbon transformations by attached bacterial populations in granitic ground water from deep crystalline bed-rock of the Stripa research mine. *Microbiology* 140 1565–1573. 10.1099/13500872-140-7-1565 8075798

[B38] Engel A. S. (2019). “Microbes,” in *Encyclopedia of Caves*, eds White W. B. Culver D. C. Pipan T. (Amsterdam: Elsevier).

[B39] Etiope G. (2017). Abiotic methane in continental serpentinization sites: an overview. *Proc. Earth Plan. Sci.* 17 9–12. 10.1016/j.proeps.2016.12.006

[B40] Etiope G. Vadillo I. Whiticar M. J. Marques J. M. Carreira P. M. Tiago I. (2016). Abiotic methane seepage in the Ronda peridotite massif, southern Spain. *Appl. Geochem.* 66 101–113. 10.1016/j.apgeochem.2015.12.001

[B41] Flemming H.-C. Wuertz S. (2019). Bacteria and archaea on Earth and their abundance in biofilms. *Nat. Rev. Microbiol.* 17 247–260. 10.1038/s41579-019-0158-9 30760902

[B42] Fones E. M. Colman D. R. Kraus E. A. Nothaft D. B. Poudel S. Rempfert K. R. (2019). Physiological adaptations to serpentinization in the Samail Ophiolite. Oman. *ISME J.* 13 1750–1762. 10.1038/s41396-019-0391-2 30872803 PMC6588467

[B43] Fones E. M. Colman D. R. Kraus E. A. Stepanauskas R. Templeton A. S. Spear J. R. (2021). Diversification of methanogens into hyperalkaline serpentinizing environments through adaptations to minimize oxidant limitation. *ISME J.* 15 1121–1135. 10.1038/s41396-020-00838-1 33257813 PMC8115248

[B44] Frank Y. A. Kadnikov V. V. Gavrilov S. N. Banks D. Gerasimchuk A. L. Podosokorskaya O. A. (2016). Stable and variable parts of microbial community in siberian deep subsurface thermal aquifer system revealed in a long-term monitoring study. *Front. Microbiol.* 7:2101. 10.3389/fmicb.2016.02101 28082967 PMC5187383

[B45] Fröhlich-Nowoisky J. Kampf C. J. Weber B. Huffman J. A. Pöhlker C. Andreae M. O. (2016). Bioaerosols in the earth system: climate, health, and ecosystem interactions. *Atmospheric Res.* 182 346–376. 10.1016/j.atmosres.2016.07.018

[B46] Fukuda A. Hagiwara H. Ishimura T. Kouduka M. Ioka S. Amano Y. (2010). Geomicrobiological properties of ultra-deep granitic groundwater from the Mizunami Underground Research Laboratory (MIU), Central Japan. *Microb. Ecol.* 60 214–225. 10.1007/s00248-010-9683-9 20473491

[B47] Gihring T. M. Moser D. P. Lin L.-H. Davidson M. Onstott T. C. Morgan L. (2006). The distribution of microbial taxa in the subsurface water of the kalahari shield, South Africa. *Geomicrobiol. J.* 23 415–430. 10.1080/01490450600875696

[B48] Glombitza C. Putman L. I. Rempfert K. R. Kubo M. D. Schrenk M. O. Templeton A. S. (2021). Active microbial sulfate reduction in fluids of serpentinizing peridotites of the continental subsurface. *Commun. Earth Environ.* 2:84. 10.1038/s43247-021-00157-z

[B49] Griffin D. W. (2008). Non-spore forming eubacteria isolated at an altitude for 20,000 m in Earth’s atmosphere: extended incubation periods needed for culture-based assays. *Aerobiologia* 24 19–25. 10.1007/s10453-007-9078-7

[B50] Hallbeck L. Pedersen K. (2008). Characterization of microbial processes in deep aquifers of the Fennoscandian Shield. *Appl. Geochem.* 23 1796–1819. 10.1016/j.apgeochem.2008.02.012

[B51] Hallbeck L. Pedersen K. (2012). Culture-dependent comparison of microbial diversity in deep granitic groundwater from two sites considered for a Swedish final repository of spent nuclear fuel. *FEMS Microbiol. Ecol.* 81 66–77. 10.1111/j.1574-6941.2011.01281.x 22188407

[B52] Hodgson D. A. Bentley M. J. Smith J. A. Klepacki J. Makinson K. Smith A. M. (2016). Technologies for retrieving sediment cores in Antarctic subglacial settings. *Philos. Trans. R. Soc. A* 374:20150056. 10.1098/rsta.2015.0056 26667918

[B53] Hu P. Tom L. Singh A. Thomas B. C. Baker B. J. Piceno Y. M. (2016). Genome-resolved metagenomic analysis reveals roles for candidate phyla and other microbial community members in biogeochemical transformations in oil reservoirs. *mBio* 7:e01669-15. 10.1128/mBio.01669-15 26787827 PMC4725000

[B54] Ionescu D. Heim C. Thiel V. Ramette A. Reitner J. de Beer D. (2015). Diversity of iron oxidizing and reducing bacteria in bioreactors set in the Äspö Hard Rock Laboratory. *Geomicrobiology* 32 207–220.

[B55] Itävaara M. Nyyssönen M. Kapanen A. Mousiainen A. Ahonen L. Kukkonen I. (2011). Characterization of bacterial diversity to a depth of 1500m in the Outokumpu deep borehole, Fennoscandian Shield. *FEMS Microbiol. Ecol.* 77 295–309. 10.1111/j.1574-6941.2011.01111.x 21488910

[B56] Jägevall S. Lisa Rabe L. Pedersen K. (2011). Abundance and diversity of biofilms in natural and artificial aquifers of the äspö hard rock laboratory, Sweden. *Microbial Ecol.* 61 410–422. 10.1007/s00248-010-9761-z 21132427

[B57] Johansson Å (1988). The age and geotectonic setting of the Småland-Värmland granite porphyry belt. *GFF* 110 105–110. 10.1080/11035898809452648

[B58] Jones D. S. Northup D. E. (2021). Cave decorating with microbes: geomicrobiology of caves. *Elements* 17 107–112. 10.2138/gselements.17.2.107

[B59] Jungbluth S. P. Glavina del Rio T. Tringe S. G. Stepanauskas R. Rappeì M. S. (2017). Genomic comparisons of a bacterial lineage that inhabits both marine and terrestrial deep subsurface systems. *PeerJ* 5:e3134. 10.7717/peerj.3134 28396823 PMC5385130

[B60] Katsuyama C. Nashimoto H. Nagaosa K. Ishibashi T. Furuta K. Kinoshita T. (2013). Occurrence and potential activity of denitrifiers and methanogens in groundwater at 140m depth in Pliocene diatomaceous mudstone of northern Japan. *FEMS Microbiol. Ecol.* 86 532–543. 10.1111/1574-6941.12179 23845087

[B61] Kay C. M. Haanela A. Johnson D. B. (2014). Microorganisms in subterranean acidic waters within Europe’s deepest metal mine. *Res. Microbiol.* 165 705–712. 10.1016/j.resmic.2014.07.007 25063488

[B62] Kelemen P. B. Matter J. M. Teagle D. A. H. Coggon J. A., and The Oman Drilling Project Science Team. (2020). “Microbiology,” in *Proceedings of the International Ocean Discovery Program*, (College Station, TX).

[B63] Kelemen P. Rajhi A. A. Godard M. Ildefonse B. Köpke J. MacLeod C. (2013). Scientific drilling and related research in the samail ophiolite, sultanate of oman. *Sci. Drilling* 15 64–71. 10.5194/sd-15-64-2013

[B64] Kieft T. L. (2016). “Microbiology of the deep continental biosphere,” in *Their World: A Diversity of Microbial Environments, Advances in Environmental Microbiology*, ed. Hurst C. J. (Berlin: Springer), 10.1007/978-3-319-28071-4_6

[B65] Kotelnikova S. Pedersen K. (1998). Distribution and activity of methanogens and homoacetogens in deep granitic aquifers at Äspö Hard Rock Laboratory, Sweden. *FEMS Microbiol. Ecol.* 26 121–134. 10.1016/s0168-6496(98)00028-2

[B66] Kotelnikova S. Macario A. J. L. Pedersen K. (1998). Methanobacterium subterraneum, a new species of Archaea isolated from deep groundwater at the Äspö Hard Rock Laboratory, Sweden. *Int. J. Systematic Bacteriol.* 48 357–367. 10.1099/00207713-48-2-357 9731274

[B67] Kraus E. A. Nothaft D. Stamps B. W. Remphert K. R. Ellison E. T. Matter J. M. (2021). Molecular evidence for an active microbial methane cycle in subsurface serpentinite-hosted groundwaters in the samail ophiolite, Oman. *Appl. Environ. Microbiol.* 87:e02068-20. 10.1128/AEM.02068-2020 PMC7783335 33127818

[B68] Kutvonen H. Rajala P. Carpén L. Bomberg M. (2015). Nitrate and ammonia as nitrogen sources for deep subsurface microorganisms. *Front. Microbiol.* 6:1079. 10.3389/fmicb.2015.01079 26528251 PMC4606121

[B69] Labonteì J. M. Field E. K. Lau M. Chivian D. Van Heerden E. Wommack K. E. (2015). Single cell genomics indicates horizontal gene transfer and viral infections in a deep subsurface Firmicutes population. *Front. Microbiol.* 6:349. 10.3389/fmicb.2015.00349 25954269 PMC4406082

[B70] LaRowe D. Amend J. (2014). “Energetic constraints on life in marine deep sediments,” in *Microbial Life of the Deep Biosphere*, eds Kallmeyer J. Wagner D. (Berlin: Springer), 326. 10.3389/fmicb.2014.00362

[B71] LaRowe D. Amend J. (2019). “Energy limits for life in the subsurface,” in *Deep Carbon*, eds Orcutt B. N. Daniel I. Dasgupta R. (Cambridge: Cambridge University Press), 585–619. 10.1017/9781108677950.019

[B72] Lau M. C. Y. Kieft T. L. Kuloyo O. Linage-Alvarez B. van Heerden E. Lindsay M. R. (2016). Syntrophy in the oligotrophic deep subsurface. *Proc. Natl. Acad. Sci. U S A.* 113 E7927–E7936. 27872277 10.1073/pnas.1612244113 PMC5150411

[B73] Lau M. C. Cameron C. Magnabosco C. Brown C. T. Schilkey F. Grim S. (2014). Phylogeny and phylogeography of functional genes shared among seven terrestrial subsurface metagenomes reveal N-cycling and microbial evolutionary relationships. *Front. Microbiol.* 5:531. 10.3389/fmicb.2014.00531 25400621 PMC4215791

[B74] Lauritzen S.-E. (2018). “Physiography of the Caves,” in *Cave Ecology*, eds Moldovan O. T. Kovač Ĺ Halse S. (Cham: Springer Nature Switzerland), 7–22. 10.1007/978-3-319-98852-8_2

[B75] Leong J. A. M. Shock E. L. (2020). Thermodynamic constraints on the geochemistry of low-temperature, continental, serpentinization-generated fluids. *Am. J. Sci.* 320 185–235. 10.2475/03.2020.01

[B76] Lin L.-H. Wang P.-L. Rumble D. Lippmann-Pipke J. Boice E. Pratt L. M. (2006a). Long-term sustainability of a high-energy, low-diversity crustral biome. *Science* 314 479–482. 10.1126/science.1127376 17053150

[B77] Lin L. H. Hall J. Onstott T. C. Gihring T. Sherwood Lollar B. Boice E. (2006b). Planktonic microbial communities associated with fracture-derived groundwater in a deep gold mine of South Africa. *Geomicrobiol. J.* 23 475–497. 10.1080/01490450600875829

[B78] Lippmann-Pipke J. Sherwood Lollar B. Niedermann S. Stroncik N. A. Naumann R. van Heerden E. (2011). Neon identifies two billion year old fluid component in the Kaapvaal Craton. *Chem. Geol.* 283 287–296. 10.1016/j.chemgeo.2011.01.028

[B79] Lollar G. S. Wart O. Telling J. Osburn M. R. Sherwood Lollar B. (2019). ‘Follow the water’: hydrogeochemical constraints on microbial investigations 2.4km below surface at the Kidd Creek Deep Fluid and Deep Life Observatory. *Geomicrobiol. J.* 36 859–872. 10.1080/01490451.2019.1641770

[B80] Magnabosco C. Lin L.-H. Dong H. Bomberg M. Ghiorse W. Stan-Lotter H. (2018). The biomass and biodiversity of the continental subsurface. *Nat. Geosci.* 11 707–717. 10.1038/s41561-018-0221-6

[B81] Magnabosco C. Ryan K. Lau M. C. Y. Kuloyo O. Sherwood Lollar B. Kieft T. L. (2016). A metagenomic window into carbon metabolism at 3 km depth in Precambrian continental crust. *ISME J.* 10 730–741. 10.1038/ismej.2015.150 26325359 PMC4817676

[B82] Magnabosco C. Tekere M. Lau M. C. Y. Linage B. Kuloyo O. Erasmus M. (2014). Comparisons of the composition and biogeographic distribution of the bacterial communities occupying South African thermal springs with those inhabiting deep subsurface fracture water. *Front. Microbiol.* 5:679. 10.3389/fmicb.2014.00679 25566203 PMC4269199

[B83] Mailloux B. J. Fuller M. E. Onstott T. C. Hall J. Dong H. DeFlaun M. F. (2003). The role of physical, chemical, and microbial heterogeneity on the field-scale transport and attachment of bacteria. *Water Resources Manag.* 39:hbox1142. 10.1029/2002WR001591

[B84] Margesin R. Miteva V. (2011). Diversity and ecology of psychrophilic microorganisms. *Res. Microbiol.* 162 346–361. 10.1016/j.resmic.2010.12.004 21187146

[B85] Martin W. Baross J. Kelley D. Russell M. J. (2008). Hydrothermal vents and the origin of life. *Nat. Rev. Microbiol.* 6 805–814. 10.1038/nrmicro1991 18820700

[B86] McCollom T. M. (2000). Geochemical constraints on primary productivity in submarine hydrothermal vent plumes. *Deep Sea Res. Part I: Oceanographic Res. Papers* 47 85–101. 10.1016/s0967-0637(99)00048-5

[B87] McCollom T. M. Bach W. (2009). Thermodynamic constraints on hydrogen generation during serpentinization of ultramafic rocks. *Geochim. Cosmochimica Acta* 73 856–875. 10.1016/j.gca.2008.10.032

[B88] McCollom T. M. Sherwood Lollar B. Lacrampe-Couloume G. Seewald J. S. (2010). The influence of carbon source on abiotic organic synthesis and carbon isotope fractionation under hydrothermal conditions. *Geochim. Cosmochimica Acta* 74 2717–2740. 10.1016/j.gca.2010.02.008

[B89] Merino N. Aronson H. S. Bojanova D. P. Feyhl-Buska J. Wong M. L. Zhang S. (2019). Living at the extremes: extremophiles and the limits of life in a planetary context. *Front. Microbiol.* 10:780. 10.3389/fmicb.2019.00780 31037068 PMC6476344

[B90] Merino N. Kawai M. Boyd E. S. Colman D. R. McGlynn S. E. Nealson K. H. (2020). Single-Cell genomics of novel actinobacteria with the wood–ljungdahl pathway discovered in a serpentinizing system. *Front. Microbiol.* 11:1031. 10.3389/fmicb.2020.01031 32655506 PMC7325909

[B91] Meyer-Dombard D. R. Casar C. P. Simon A. G. Cardace D. Schrenk M. O. Arcilla C. A. (2018). Biofilm formation and potential for iron cycling in serpentinization-influenced groundwater of the Zambales and Coast Range ophiolites. *Extremophiles* 22 407–431. 10.1007/s00792-018-1005-z 29450709

[B92] Meyer-Dombard D. R. Osburn M. R. Cardace D. Arcilla C. A. (2019). The effect of a tropical climate on available nutrient resources to springs in ophiolite-hosted, deep biosphere ecosystems in the Philippines. *Front. Microbiol.* 10:761. 10.3389/fmicb.2019.00761 31118921 PMC6504838

[B93] Meyer-Dombard D. R. Woycheese K. M. Yargıçoğlu, Cardace D. Shock E. L. Güleçal-Pektas (2015). High pH microbial ecosystems in a newly discovered, ephemeral, serpentinizing fluid seep at Yanastas (Chimera). *Front. Microbiol.* 5:723. 10.3389/fmicb.2014.00723 25646094 PMC4298219

[B94] Miettinen H. Bomberg M. Vikman M. (2018). Acetate activates deep subsurface fracture fluid microbial communities in Olkiluoto, Finland. *Geosciences* 8:399. 10.3390/geosciences8110399

[B95] Miettinen H. Kietäväinen R. Sohlberg E. Numminen M. Ahonen L. Itävaara M. (2015). Microbiome composition and geochemical characteristics of deep subsurface high-pressure environment, Pyhäsalmi mine Finland. *Front. Microbiol.* 6:1203. 10.3389/fmicb.2015.01203 26579109 PMC4626562

[B96] Miller H. M. Chaudhry N. Conrad M. E. Bill M. Kopf S. H. Templeton A. S. (2018). Large carbon isotope variability during methanogenesis under alkaline conditions. *Geochim. Cosmochimica Acta* 237 18–31. 10.1016/j.gca.2018.06.007

[B97] Miller H. M. Matter J. M. Kelemen P. Ellison E. T. Conrad M. E. Fierer N. (2016). Modern water/rock reactions in Oman hyperalkaline peridotite aquifers and implications for microbial habitability. *Geochim. Cosmochimica Acta* 179 217–241. 10.1016/j.gca.2016.01.033

[B98] Miteva V. Burlingame C. Sowers T. Brenchley J. (2014). Comparative evaluation of the indigenous microbial diversity vs. drilling fluid contaminants in the NEEM Greenland ice core. *FEMS Microbiol. Ecol.* 89 238–256. 10.1111/1574-6941.12286 24450335

[B99] Miteva V. Sowers T. Schüpbach S. Fischer H. Brenchley J. (2016). Geochemical and microbiological studies of nitrous oxide variations within the new NEEM Greenland ice core during the last glacial period. *Geomicrobiol. J.* 33 647–660. 10.1080/01490451.2015.1074321

[B100] Momper L. Kiel Reese B. Zinke L. Wanger G. Osburn M. R. Moser D. (2017a). Major phylum-level differences between porefluid and host rock bacterial communities in the terrestrial deep subsurface. *Environ. Microbiol. Rep.* 9 501–511. 10.1111/1758-2229.12563 28677247

[B101] Momper L. Jungbluth S. P. Lee M. D. Amend J. P. (2017b). Energy and carbon metabolisms in a deep terrestrial subsurface fluid microbial community. *ISME J.* 11 2319–2333. 10.1038/ismej.2017.94 28644444 PMC5607374

[B102] Moody J. B. (1976). Serpentinization: a review. *Lithos* 9 125–138. 10.1016/0024-4937(76)90030-x

[B103] Morono Y. Inagaki F. (2016). Analysis of low-biomass microbial communities in the deep biosphere. *Adv. Appl. Microbiol.* 95 149–178. 10.1016/bs.aambs.2016.04.001 27261783

[B104] Morrill P. L. Kuenen J. G. Johnson O. J. Suzuki S. Rietze A. Sessions A. L. (2013). Geochemistry and geobiology of a present-day serpentinization site in California: the Cedars. *Geochim. Cosmochimica Acta* 109 222–240. 10.1016/j.gca.2013.01.043

[B105] Moser D. P. Gihring T. M. Brockman F. J. Fredrickson J. K. Balkwill D. L. Dollhopf M. E. (2005). *Desulfotomaculum* and *Methanobacterium spp*. Dominate a 4- to 5-Kilometer-Deep Fault. *Appl. Environ. Microbiol.* 71 8773–8783. 10.1128/AEM.71.12.8773-8783.2005 16332873 PMC1317344

[B106] Moser D. P. Onstott T. C. Fredrickson J. K. Brockman F. J. Balkwill D. L. Drake G. R. (2003). Temporal shifts in the geochemistry and microbial community structure of an ultradeep mine borehole following isolation. *Geomicrobiol. J.* 20 517–548. 10.1080/713851170

[B107] Motamedi M. Pedersen K. (1998). Desulfovibrio aespoeensis sp. nov. a mesophilic sulfate-reducing bacterium from deep groundwater at Äspö Hard Rock Laboratory, Sweden. *Int. J. Systematic Bacteriol.* 48 311–315. 10.1099/00207713-48-1-311 9542102

[B108] Neal C. Stanger G. (1983). Hydrogen generation from mantle source rocks in oman. *Earth Plan. Sci. Lett.* 66 315–320. 10.1016/0012-821x(83)90144-9

[B109] Nicolaysen L. O. Hart R. J. Gale N. H. (1981). The Vredefort radioelement profile extended to supracrustal strata at Carletonville, with implications for continental heat flow. *J. Geophys. Res.* 86 10653–10661. 10.1029/jb086ib11p10653

[B110] Nothaft D. B. Templeton A. S. Boyd E. S. Matter J. M. Stute M. Paukert Vankeuren A. N. (2021a). Aqueous geochemical and microbial variation across discrete depth intervals in a peridotite aquifer assessed using a packer system in the samail ophiolite, oman. *J. Geophys. Res. Biogeosci.* 126:e2021JG006319. 10.1029/2021jg006319

[B111] Nothaft D. B. Templeton A. S. Rhim J. H. Wang D. T. Labidi J. Miller H. M. (2021b). Geochemical, biological, and clumped isotopologue evidence for substantial microbial methane production under carbon limitation in serpentinites of the samail ophiolite, oman. *J. Geophys. Res. Biogeosci.* 126:e2020JG006025. 10.1029/2020jg006025

[B112] Nuppunen-Puputti M. Purkamo L. Kietäväinen R. Nyyssönen M. Itävaara M. Ahonen L. (2018). Rare biosphere Archaea assimilate acetate in *Precambrian terrestrial* subsurface at 2.2 km depth. *Geosciences* 8:418. 10.3390/geosciences8110418

[B113] Nyyssönen M. Bomberg M. Kapanen A. Nousiainen A. Pitkänen P. Itävaara M. (2012). Methanogenic and sulphate-reducing microbial communities in deep groundwater of crystalline rock fractures in Olkiluoto, Finland. *Geomicrobiol. J.* 29 863–878. 10.1080/01490451.2011.635759

[B114] Nyyssönen M. Hultman J. Ahonen L. Kukkonen I. Paulin L. Laine P. (2014). Taxonomically and functionally diverse microbial communities in deep crystalline rocks of the Fennoscandian shield. *ISME J.* 8 126–138. 10.1038/ismej.2013.125 23949662 PMC3869007

[B115] Osburn M. R. Kruger B. Masterson A. L. Casar C. P. Amend J. P. (2019). Establishment of the deep mine microbial observatory (DeMMO), South Dakota, USA, a geochemically stable portal into the deep subsurface. *Front. Earth Sci.* 7:196. 10.3389/feart.2019.00196

[B116] Osburn M. R. LaRowe D. E. Momper L. M. Amend J. P. (2014). Chemolithotrophy in the continental deep subsurface: Sanford Underground Research Facility (SURF), USA. *Front. Microbiol.* 5:610. 10.3389/fmicb.2014.00610 25429287 PMC4228859

[B117] Parkes R. J. Cragg B. Roussel E. Webster G. Weightman A. Sass H. (2014). A review of prokaryotic populations and processes in sub-seafloor sediments, including biosphere:geosphere interactions. *Mar. Geol.* 352 409–425. 10.1016/j.margeo.2014.02.009

[B118] Payler S. J. Biddle J. F. Sherwood Lollar B. Fox-Powell M. G. Edwards T. Ngwenya B. T. (2019). An ionic limit to life in the deep subsurface. *Front. Microbiol.* 10:426. 10.3389/fmicb.2019.00426 30915051 PMC6422919

[B119] Pearce D. A. Bridge P. D. Hughes K. A. Sattler B. Psenner R. Russell N. J. (2009). Microorganisms in the atmosphere over Antarctica. *FEMS Microbiol. Ecol.* 69 143–157. 10.1111/j.1574-6941.2009.00706.x 19527292

[B120] Pedersen K. (1993). Bacterial processes in nuclear waste disposal. *Microbiol. Eur.* 1 18–23.

[B121] Pedersen K. (2012). Subterranean microbial populations metabolize hydrogen and acetate under *in situ* conditions in granitic groundwater at 450 m depth in the Äspö Hard Rock Laboratory, Sweden. *FEMS Microbiol. Ecol.* 81 217–229. 10.1111/j.1574-6941.2012.01370.x 22452510

[B122] Pedersen K. (2013). Metabolic activity of subterranean microbial communities in deep granitic groundwater supplemented with methane and H2. *ISME J.* 7 839–849. 10.1038/ismej.2012.144 23235288 PMC3603388

[B123] Pedersen K. Ekendahl S. (1992). Incorporation of CO2 and introduced organic compounds by bacterial populations in groundwater from the deep crystalline bedrock of the Stripa mine. *J. General Microbiol.* 138 369–376. 10.1099/00221287-138-2-369

[B124] Preiner M. Xavier J. C. Sousa F. L. Zimorski V. Neubeck A. Lang S. Q. (2018). Serpentinization: connecting geochemistry, ancient metabolism and industrial hydrogenation. *Life-Basel* 8:41. 10.3390/life8040041 30249016 PMC6316048

[B125] Price P. B. (2007). Microbial life in glacial ice and implications for a cold origin of life. *FEMS Microbiol. Ecol.* 59 217–231. 10.1111/j.1574-6941.2006.00234.x 17328118

[B126] Purkamo L. Bomberg M. Nyyssönen M. Kukkonen I. Ahonen L. Itävaara M. (2015a). Heterotrophic communities supplied by ancient organic carbon predominate in deep Fennoscandian bedrock fluids. *Microb. Ecol.* 69 319–332. 10.1007/s00248-014-0490-6 25260922

[B127] Purkamo L. Bomberg M. Kietäväinen R. Salavirta H. Nyyssönen M. Nuppunen-Puputti M. (2015b). The keystone species of Precambrian deep bedrock biosphere belong to *Burkholderiales* and *Clostridiales*. *Biogeosci. Discussions* 12 18103–18150.

[B128] Purkamo L. Bomberg M. Kietäväinen R. Salavirta H. Nyyssönen M. Nuppunen-Puputti M. (2016). Microbial co-occurrence patterns in deep Precambrian bedrock fracture fluids. *Biogeosciences* 13 3091–3108. 10.5194/bg-13-3091-2016

[B129] Purkamo L. Bomberg M. Nyyssönen M. Ahonen L. Kukkonen I. Itävaara M. (2017). Response of deep subsurface microbial community to different carbon sources and electron acceptors during ∼2 months incubation in microcosms. *Front. Microbiol.* 8:232. 10.3389/fmicb.2017.00232 28265265 PMC5316538

[B130] Purkamo L. Bomberg M. Nyyssönen M. Kukkonen I. Ahonen L. Kietäväinen R. (2013). Dissecting the deep biosphere: retrieving authentic microbial communities from packer-isolated deep crystalline bedrock fracture zones. *FEMS Microbiol. Ecol.* 85 324–337. 10.1111/1574-6941.12126 23560597

[B131] Purkamo L. Kietäväinen R. Miettinen H. Sohlberg E. Kukkonen I. Itavaara M. (2018). Diversity and functionality of archaeal, bacterial and fungal communities in deep Archaean bedrock groundwater. *FEMS Microbiol. Ecol.* 94:fiy116. 10.1093/femsec/fiy116 29893836

[B132] Purkamo L. Kietäväinen R. Nuppunen-Puputti M. Bomberg M. Cousins C. (2020). Ultradeep microbial communities at 4.4 km within crystalline bedrock: implications for habitability in a planetary context. *Life* 10:2. 10.3390/life10010002 31947979 PMC7175195

[B133] Putman L. I. Sabuda M. C. Brazelton W. J. Kubo M. D. Hoehler T. M. McCollom T. M. (2021). Microbial communities in a serpentinizing aquifer are assembled through strong concurrent dispersal limitation and selection. *mSystems* 6:e0030021. 10.1128/mSystems.00300-21 34519519 PMC8547479

[B134] Quéméneur M. Palvadeau A. Postec A. Monnin C. Chavagnac V. Ollivier B. (2015). Endolithic microbial communities in carbonate precipitates from serpentinite-hosted hyperalkaline springs of the *Voltri Massif* (Ligurian Alps, Northern Italy). *Environ. Sci. Pollution Res. Int.* 22 13613–13624. 10.1007/s11356-015-4113-7 25874424

[B135] Rempfert K. R. Miller H. M. Bompard N. Nothaft D. Matter J. M. Kelemen P. (2017). Geological and geochemical controls on subsurface microbial life in the samail ophiolite, Oman. *Front. Microbiol.* 8:56. 10.3389/fmicb.2017.00056 28223966 PMC5293757

[B136] Rhodes R. H. Faïn X. Stowasser C. Blunier T. Chappelaz J. McConnell J. R. (2013). Continuous methane measurements from a late Holocene Greenland ice core: atmospheric and in situ signals. *Earth Plan. Sci. Lett.* 368 9–19. 10.1016/j.epsl.2013.02.034

[B137] Rowe A. R. Abuyen K. Lam B. R. Kruger B. Casar C. P. Osburn M. R. (2020). Electrochemical evidence for in situ microbial activity at the Deep Mine Microbial Observatory (DeMMO), South Dakota, USA. *Geobiology* 19 173–188. 10.1111/gbi.12420 33188587

[B138] Rowe A. R. Yoshimura M. LaRowe D. E. Bird L. J. Amend J. P. Hashimoto K. (2017). In situ electrochemical enrichment and isolation of a magnetite-reducing bacterium from a high pH serpentinizing spring. *Environ. Microbiol.* 19 2272–2285. 10.1111/1462-2920.13723 28276203

[B139] Russell M. J. Ponce A. (2020). Six ‘Must-Have’ minerals for life’s emergence: olivine, pyrrhotite, bridgmanite, serpentine, fougerite and mackinawite. *Life-Basel* 10:291. 10.3390/life10110291 33228029 PMC7699418

[B140] Sabuda M. C. Brazelton W. J. Putman L. I. McCollom T. M. Hoehler T. M. Kubo M. D. Y. (2020). A dynamic microbial sulfur cycle in a serpentinizing continental ophiolite. *Environ. Microbiol.* 22 2329–2345. 10.1111/1462-2920.15006 32249550

[B141] Sabuda M. C. Putman L. I. Hoehler T. M. Kubo M. D. Brazelton W. J. Cardace D. (2021). Biogeochemical gradients in a serpentinization-influenced aquifer: implications for gas exchange between the subsurface and atmosphere. *J. Geophys. Res. Biogeosci.* 126:e2020JG006209. 10.1029/2020jg006209

[B142] Sahl J. W. Schmidt R. Swanner E. D. Mandernack K. W. Templeton A. S. Kieft T. L. (2008). Subsurface microbial diversity in deep granitic fracture water in Colorado. *Appl. Environ. Microbiol.* 74 143–152. 10.1128/AEM.01133-07 17981950 PMC2223202

[B143] Sánchez-Murillo R. Gazel E. Schwarzenbach E. M. Crespo-Medina M. Schrenk M. O. Boll J. (2014). Geochemical evidence for active tropical serpentinization in the Santa Elena Ophiolite, Costa Rica: an analog of a humid early Earth? *Geochem. Geophys. Geosystems* 15 1783–1800. 10.1002/2013gc005213

[B144] Schrenk M. O. Brazelton W. J. Lang S. Q. (2013). Serpentinization, Carbon, and deep life. *Rev. Mineral. Geochem.* 75 575–606. 10.2138/rmg.2013.75.18

[B145] Schubotz F. Meyer-Dombard D. R. Bradley A. S. Fredricks H. F. Hinrichs K.-U. Shock E. L. (2013). Spatial and temporal variability of biomarkers and microbial diversity reveal metabolic and community flexibility in Streamer Biofilm Communities in the Lower Geyser Basin, Yellowstone National Park. *Geobiology* 11 549–569. 10.1111/gbi.12051 23981055

[B146] Schulte M. Blake D. Hoehler T. McCollom T. (2006). Serpentinization and its implications for life on the early Earth and Mars. *Astrobiology* 6 364–376. 10.1089/ast.2006.6.364 16689652

[B147] Schulze-Makuch D. Airo A. Schirmack J. (2017). The adaptability of life on earth and the diversity of planetary habitats. *Front. Microbiol.* 8:2011. 10.3389/fmicb.2017.02011 29085352 PMC5650640

[B148] Schwarzenbach E. M. Früh-Green G. L. Bernasconi S. M. Alt J. C. Shanks Iii W. C. Gaggero L. (2012). Sulfur geochemistry of peridotite-hosted hydrothermal systems: comparing the Ligurian ophiolites with oceanic serpentinites. *Geochim. Cosmochimica Acta* 91 283–305. 10.1016/j.gca.2012.05.021

[B149] Selensky M. J. Masterson A. L. Blank J. G. Lee S. C. Osburn M. R. (2021). Stable carbon isotope depletions in lipid biomarkers suggest subsurface carbon fixation in lava caves. *J. Geophys. Res. Biogeosci.* 126:e2021JG006430. 10.1029/2021jg006430

[B150] Sherwood Lollar B. Frape S. K. Fritz P. Macko S. A. Welhan J. A. Blomqvist R. (1993). Evidence for bacterially generated hydrocarbon gas in Canadian shield and Fennoscandian shield rocks. *Geochim. Cosmochimica Acta* 57 5073–5085. 10.1016/0016-7037(93)90609-z

[B151] Sherwood Lollar B. Voglesonger K. Lin L. H. Lacrampe-Couloume G. Telling J. Abrajano T. A. (2007). Hydrogeologic controls on episodic H2 release from Precambrian fractured rocks: energy for deep subsurface life on Earth and Mars. *Astrobiology* 7 971–986. 10.1089/ast.2006.0096 18163873

[B152] Shock E. Canovas P. (2010). THe potential for abiotic organic synthesis and biosynthesis at seafloor hydrothermal systems. *Geofluids* 10 161–192. 10.1002/9781444394900.ch12

[B153] Silver B. J. Raymond R. Sigman D. Prokopeko M. Lollar B. Lacrampe-Couloume G. (2012). The origin of NO3− and N2 in deep subsurface fracture water of South Africa. *Chem. Geol.* 294-295 51–62. 10.1016/j.chemgeo.2011.11.017

[B154] Simkus D. N. Slater G. F. Lollar B. S. Wilkie K. Kieft T. L. Magnabosco C. (2016). Variations in microbial car- bon sources and cycling in the deep continental subsurface. *Geochim. Cosmochimica Acta* 173 264–283. 10.1016/j.gca.2015.10.003

[B155] Sojo V. Herschy B. Whicher A. Camprubi E. Lane N. (2016). The origin of life in alkaline hydrothermal vents. *Astrobiology* 16 181–197. 10.1089/ast.2015.1406 26841066

[B156] Spear J. R. Barton H. A. Francis C. A. Roberts K. J. Pace N. R. (2007). Microbial community biofabrics in a geothermal mine adit. *Appl. Environ. Microbiol.* 73 6172–6180. 10.1128/AEM.00393-07 17693567 PMC2075011

[B157] Stevens T. O. McKinley J. P. (1995). Lithoautotrophic microbial ecosystems in deep basalt aquifers. *Science* 270 450–454. 10.1038/s41467-017-01288-8 29051484 PMC5648843

[B158] Stroes-Gascoyne S. Schippers A. Schwyn B. Poulain S. Sergeant C. Simonoff M. (2007). Microbial community analysis of Opalinus Clay drill core samples from the Mont Terri underground research laboratory, Switzerland. *Geomicrobiol. J.* 24 1–17. 10.1080/01490450601134275

[B159] Suzuki S. Ishii S. Hoshino T. Rietze A. Tenney A. Morrill P. L. (2017). Unusual metabolic diversity of hyperalkaliphilic microbial communities associated with subterranean serpentinization at the Cedars. *ISME J.* 11 2584–2598. 10.1038/ismej.2017.111 28731475 PMC5649167

[B160] Suzuki S. Ishii S. Wu A. Cheung A. Tenney A. Wagner G. (2013). Microbial diversity in The Ceders, an ultrabasic, ultrareducing, and low salinity serpentinizing ecosystem. *Proc. Natl. Acad. Sci. U S A.* 110 15336–15341. 10.1073/pnas.1302426110 24003156 PMC3780913

[B161] Suzuki S. Nealson K. H. Ishii S. (2018). Genomic and in-situ transcriptomic characterization of the candidate phylum NPL-UPL2 from highly alkaline highly reducing serpentinized groundwater. *Front. Microbiol.* 9:3141. 10.3389/fmicb.2018.03141 30619209 PMC6305446

[B162] Swanner E. D. Templeton A. S. (2011). Potential for nitrogen fixation and nitrification in the granite-hosted subsurface at Henderson Mine, CO. *Front. Microbiol.* 2:254. 10.3389/fmicb.2011.00254 22190904 PMC3243026

[B163] Swingley W. D. Meyer-Dombard D. R. Shock E. L. Alsop E. B. Falenski H. D. Havig J. R. (2012). Coordinating environmental genomics and geochemistry reveals metabolic transitions in a hot spring ecosystem. *PLoS One* 7:e38108. 10.1371/journal.pone.0038108 22675512 PMC3367023

[B164] Taubner R. S. Olsson-Francis K. Vance S. D. Ramkissoon N. K. Postberg F. de Vera J. P. (2020). Experimental and simulation efforts in the astrobiological exploration of exooceans. *Space Sci. Rev.* 216:9. 10.1007/s11214-020-0635-5 32025060 PMC6977147

[B165] Templeton A. S. Ellison E. T. Glombitza C. Morono Y. Rempfert K. R. Hoehler T. M. (2021). Accessing the subsurface biosphere within rocks undergoing active low-temperature serpentinization in the samail ophiolite (Oman Drilling Project). *J. Geophys. Res. Biogeosci.* 126:e2021JG006315. 10.1029/2021jg006315

[B166] Thieringer P. H. Honeyman A. S. Spear J. R. (2021). Spatial and temporal constraints on the composition of microbial communities in subsurface boreholes of the Edgar Experimental Mine. *Microbiol. Spectrum.* 9:e00631-21. 10.1128/Spectrum.00631-21 34756066 PMC8579930

[B167] Tiago I. Veriìssimo A. (2013). Microbial and functional diversity of a subterrestrial high pH groundwater associated to serpentinization. *Environ. Microbiol.* 15 1687–1706. 10.1111/1462-2920.12034 23731249

[B168] Tiago I. Chung A. P. Verissimo A. (2004). Bacterial diversity in a nonsaline alkaline environment: heterotrophic aerobic populations. *Appl. Environ. Microbiol.* 70 7378–7387. 10.1128/AEM.70.12.7378-7387.2004 15574939 PMC535156

[B169] Tung H. C. Bramall N. E. Price P. B. (2005). Microbial origin of excess methane in glacial ice and implications for life on Mars. *Proc. Natl. Acad. Sci. U S A.* 102 18292–18296. 10.1073/pnas.0507601102 16339015 PMC1308353

[B170] Tung H. C. Price P. B. Bramall N. E. Vrdoljak G. (2006). Microorganisms metabolizing on clay grains in 3-km-deep Greenland basal ice. *Astrobiology* 6 69–86. 10.1089/ast.2006.6.69 16551227

[B171] Twing K. I. Brazelton W. J. Kubo M. D. Hyer A. J. Cardace D. Hoehler T. M. (2017). Serpentinization-Influenced groundwater harbors extremely low diversity microbial communities adapted to high pH. *Front. Microbiol.* 8:308. 10.3389/fmicb.2017.00308 28298908 PMC5331062

[B172] Vaïtilingom M. Deguillaume L. Vinatier V. Sancelme M. Amato P. Chaumerliac N. (2013). Potential impact of microbial activity on the oxidant capacity and organic carbon budget in clouds. *Proc. Natl. Acad. Sci. U S A.* 110 559–564. 10.1073/pnas.1205743110 23263871 PMC3545818

[B173] Wainwright M. Wickramasinghe N. Narlikar J. Rajaratnam P. (2004). Microorganisms cultured from stratospheric air samples obtained at 41 km. *FEMS Microbiol. Lett.* 218 161–165. 10.1111/j.1574-6968.2003.tb11513.x 12583913

[B174] Wanger G. Southam G. Onstott T. C. (2006). Structural and chemical characterization of a natural fracture surface from 2.8 kilometers below land surface: biofilms in the deep subsurface. *Geomicrobiol. J.* 23 443–452. 10.1080/01490450600875746

[B175] Ward J. A. Slater G. F. Moser D. P. Lin L.-H. Lacrampe-Couloume G. Bonin A. S. (2004). Microbial hydrocarbon gasses in the Witwatersrand Basin, South Africa: implications for the deep biosphere. *Geochim. Cosmochimica Acta* 68 3239–3250. 10.1016/j.gca.2004.02.020

[B176] Woycheese K. M. Meyer-Dombard D. R. Cardace D. Argayosa A. M. Arcilla C. A. (2015). Out of the dark: transitional subsurface-to-surface microbial diversity in a terrestrial serpentinizing seep (Manleluag, Pangasinan, the Philippines). *Front. Microbiol.* 6:44. 10.3389/fmicb.2015.00044 25745416 PMC4333863

[B177] Wu X. Holmfeldt K. Hubalek V. Lundin D. Åström M. Bertilsson S. (2016). Microbial metagenomes from three aquifers in the Fennoscandian shield terrestrial deep biosphere reveal metabolic partitioning among populations. *ISME J.* 10 1192–1203. 10.1038/ismej.2015.185 26484735 PMC5029217

[B178] Zhang G. Dong H. Xu Z. Zhao D. Zhang C. (2005). Microbial diversity in ultra-high-pressure rocks and fluids from the chinese continental scientific drilling project in China. *Appl. Environ. Microbiol.* 71 3213–3227. 10.1128/AEM.71.6.3213-3227.2005 15933024 PMC1151863

[B179] Zhong Z.-P. Solonenko N. E. Gazituìa M. C. Kenny D. V. Mosley-Thompson E. Rich V. I. (2018). Clean low-biomass procedures and their application to ancient ice core microorganisms. *Front. Microbiol.* 9:1094. 10.3389/fmicb.2018.01094 29910780 PMC5992382

[B180] Zhong Z.-P. Tian F. Roux S. Gazitúa M. C. Solonenko N. E. Li Y.-F. (2021). Glacier ice archives nearly 15,000-year-old microbes and phages. *Microbiome* 9:160. 10.1186/s40168-021-01106-w 34281625 PMC8290583

